# AI-driven cybersecurity framework for software development based on the ANN-ISM paradigm

**DOI:** 10.1038/s41598-025-97204-y

**Published:** 2025-04-18

**Authors:** Habib Ullah Khan, Rafiq Ahmad Khan, Hathal S. Alwageed, Alaa Omran Almagrabi, Sarra Ayouni, Mohamed Maddeh

**Affiliations:** 1 Department of Accounting and Information Systems, College of Bussiness and Economics, Qatar University, Doha, Qatar; 2 Software Engineering Research Group, Department of Computer Science and IT, University of Malakand, Chakdara, Pakistan; 3 College of Computer and Information Sciences, Jouf University, 42421 Sakaka, Saudi Arabia; 4 Department of Information Systems, Faculty of Computing and Information Technology, King Abdulaziz University, Jeddah, Saudi Arabia; 5 Department of Information Systems, College of Computer and Information Sciences, Princess Nourah Bint Abdulrahman University, P.O. Box 84428, 11671 Riyadh, Saudi Arabia; 6 College of Applied Computer Science, King Saud University, 11451 Riyadh, Saudi Arabia

**Keywords:** AI, Secure software coding, Cybersecurity risks and practices, Systematic literature review, Empirical survey, Case study, ANN-ISM modeling, Cybersecurity maturity levels, Engineering, Mathematics and computing

## Abstract

With the increasing reliance on software applications, cybersecurity threats have become a critical concern for developers and organizations. The answer to this vulnerability is AI systems, which help us adapt a little better, as traditional measures in security have failed to respond to the upcoming threats. This paper presents an innovative cybersecurity framework using AI, by the Artificial Neural Network (ANN)—Interpretive Structural Modeling (ISM) model, to improve threat detection, vulnerability assessment, and risk response during software development. This framework helps realize dynamic, intelligent security as a part of the Software Development life cycle (SDLC). Initially, existing cybersecurity risks in software coding are systematically evaluated to identify potential gaps and integrate best practices into the proposed model. In the second phase, an empirical survey was conducted to identify and validate the findings of the systematic literature review (SLR). In the third phase, a hybrid approach is employed, integrating ANN for real-time threat detection and risk assessment. It utilizes ISM to analyze the relationships between cybersecurity risks and vulnerabilities, creating a structured framework for understanding interdependencies. A case study was conducted in the last stage to test and evaluate the AI-driven cybersecurity Mitigation Model for Secure Software Coding. A multi-level categorization system is also used to assess maturity across five key levels: Ad hoc, Planned, Standardized, Metrics-Driven, and Continuous Improvements. This study identifies 15 cybersecurity risks and vulnerabilities in software coding, along with 158 AI-driven best practices for mitigating these risks. It also identifies critical areas of insecure coding practices and develops a scalable model to address cybersecurity risks across different maturity levels. The results show that AI outperforms traditional systems in detecting security weaknesses and simultaneously fixing problems. During Levels 1–3 of the system improvement process, advanced security methods are used to protect against threats. Our analysis reveals that organizations at Levels 4 and 5 still need to entirely shift to using AI-based protection tools and techniques. The proposed system provides developers and managers with valuable insights, enabling them to select security enhancements tailored to their organization's development stages. It supports automated threat analysis, helping organizations stay vigilant against potential cybersecurity threats. The study introduces a novel ANN-ISM framework integrating AI tools with cybersecurity modeling formalisms. By merging AI systems with secure software coding principles, this research enhances the connection between AI-generated insights and real-world cybersecurity usage.

## Introduction

Nowadays, reliable software systems are critical in the functioning of all global sectors, such as healthcare, banking, finance, transportation, defense, etc. However, the ever-growing dependence on interconnected systems has made us more susceptible to cybersecurity threats. One can cause a loss of money and foul the public image^1^. Companies need to emphasize secure coding practices so that their systems are resilient to attack by malicious actors^2^. Traditional cyber defense has lost its effectiveness since conventional cyber threats have become more advanced and necessitate more competent protective measures^3^. This requires new approaches that exploit the capabilities of Artificial Intelligence (AI) to improve cybersecurity^4^.

This leads to an ever-growing range of cybersecurity concerns faced by companies undergoing digital transformation—everything from mistakes made in coding that expose systems to hacked CI/CD pipelines to poor logging and monitoring data collection^1,5,6^. These security risks are presented with bugs like race conditions, insecure authentication, and incorrectly defined security^7^. As AI matures, the convergence of technologies like cloud services and Internet of Things (IoT) devices adds layers to security challenges. Though these innovations have significant benefits to offer, they also create new vulnerabilities that organizations must proactively address using intelligent systems that are capable of responding quickly to potential threats.

Now, AI helps cybersecurity with systems capable of identifying threats instantly, reacting to them, and imagining future threats^8,9^. These systems leverage ML, NLP, and neural networks to examine the potential vulnerabilities and mitigate these threats before they get activated^9,10^. AI systems analyzing code can identify security vulnerabilities, while other AI systems also monitor how computers act to seek out potential threats^11^. While promising, AI utilization for secure software design remains immature, especially regarding established frameworks combining actionable AI using a systems approach to cybersecurity.

The study's primary objectives are identifying key cybersecurity risks by studying and ranking cybersecurity weaknesses at various software development stages using modern IT defenses. Additionally, we aim to develop AI-enabled solutions tailored to specific organizational maturity levels. Our research also seeks to create a Hybrid ANN-ISM Framework that combines ANN's pattern-recognition capabilities with ISM's hierarchical structuring abilities. Finally, we intend to enhance security maturity by establishing a roadmap for organizations to progress from basic security practices to advanced and continuous improvement stages.

This study focuses on secure software coding practices within organizations of varying maturity levels. It examines five key categories of AI-driven cybersecurity mitigation, aligned with maturity levels ranging from Ad hoc/Uncontrolled to Continuous Improvement. It evaluates practical use cases of AI applications in threat detection, automated patch management, secure development lifecycle integration, and real-time monitoring. The proposed framework aims to bridge the gap between theoretical AI models and their real-world implementation in cybersecurity.

### Comparison of the proposed framework with other existing techniques

This research assesses how our AI approach to software security development through ANN-ISM compares to regular methods across core techniques, expandability, functionality, security qualities, real-time protection, and total expense management.

- *Core approach*: The ANN-ISM paradigm links information security management with an Artificial Neural Network to spot and regulate cyber threats successfully. The ANN system uses recent and past data to recognize security dangers, while the ISM framework maintains policies for running security tasks in a planned method. These integrated elements enable the system to increase its security capabilities as new hazards emerge. Signature-based detection systems work with known threat patterns, but they cannot find newly discovered security weaknesses. In anomaly-based detection, statistics spot unusual computer activity but produce many incorrect detections during dynamic system operations. Regular machine learning systems, including decision trees and support vector machines, provide threat identification, but they need considerable data input and can fail during unconventional cyberattacks.
- *Scalability*: Using neural networks lets ISM systems handle a more significant data load when it grows. ISM framework enables security operations to expand their capabilities while sustaining changes in security risks. A signature-based system fails to handle big networks since virus updates limit effectiveness. The system detects real-time threats well, yet performance slows down in large-scale computing. Machine learning algorithms expand effectively yet need large processing equipment, mainly for database training sessions.
- *Performance*: Our proposed model uses an ANN structure to learn and adjust its security performance against the latest threats. Our system fits right into the ISM platform to keep security efforts aligned with company targets. Detectors that use signatures protect against known threats quickly yet struggle to detect emerging cyber threats effectively. The system slows down from detecting strange patterns because it has to monitor every incoming digital data. Machine learning algorithms perform better at recognizing fresh threats, but they need to process natural data patterns, so their performance decreases under specific circumstances.
- *Adaptability*: The ANN-ISM model stands out because it quickly adjusts to threats. The neural network uses incoming data to improve its security performance as security threats develop. The ISM framework helps maintain quality security procedures through regular enhancements and system updates. The system cannot automatically detect fresh dangers until someone updates the signature database, which may take too long to be helpful. The detection system finds more normal activities as new data enters the system while showing higher error rates before achieving complete learning. While machine learning systems show increased flexibility over signature-based detection, they need specific training procedures that take time and many data resources to complete.
- *Security capabilities*: Predictive analytics and advanced security controls work together within an AI framework to identify and manage potential risks. It helps to track future threats while detecting weak points to initiate security steps through various protection layers. The system finds known threats but cannot discover new attack techniques. This detection method finds unrecognized attacks but cannot handle existing security challenges quickly. When appropriately trained, machine-learning models find specific attack patterns and forecast future threats, but they can miss complex assaults.
- *Real-time response*: The ANN-ISM defense approach gives immediate response capabilities by integrating AI threat recognition with process management technology. To defend against current threats, the system takes action straightaway. Logo identification protects known hazards quickly yet cannot respond to attacks the system has never seen before. Real-time anomaly detection is slow because it needs to monitor trends constantly, while machine learning requires training updates that affect immediate security reactions.
- *Cost and maintenance*: The setup of ANN-ISM technology demands more significant investments because integrating neural networks with ISM requires expertise that comes at a higher price. Over time, the model proves more beneficial because it needs less human involvement while strengthening security and lowering incident-handling expenses. Programs based on signatures need fewer setup resources but demand more money because signature databases need regular upgrades. Monitoring and adjusting anomaly-based systems use many resources, which makes them costly for dynamic environments. Machine learning models need significant startup expenses because they require data preparation combined with software development and system updates.
- *Conclusion*: The "AI-driven Cybersecurity Framework for Software Development Based on the ANN-ISM Paradigm" provides a strong and detailed system for securing software. This hybrid approach lets AI predict threats while ISM structures monitor and defend our systems better. The model takes more resources to put into place but generates significant advantages that help it handle growing security threats over time. Old detection systems based on signatures and anomalies do not respond well to increasing security risks because they lack general flexibility. ANN-ISM provides better protection for modern software security management than alternative methods do.

The remainder of the paper is organized as follows: section “Literature review” presents a critical analysis of existing studies on cybersecurity risks, vulnerabilities, and AI applications in cybersecurity and secure software coding. Section “Research methodology” explains the ANN-ISM framework and the processes to analyze and mitigate cybersecurity risks and vulnerabilities. Section “Results and discussions” discusses the results detailing the hierarchical structure of cybersecurity risks and AI mitigation strategies. Section “Development of AI-driven cybersecurity mitigation model for secure software coding: using ANN-ISM approach” presents the proposed AI-Driven Cybersecurity Mitigation Model for Secure Software Coding (i.e., a framework using ANN-ISM). Finally, section “Implications of the study” concludes the paper by summarizing the findings, discussing the contributions of the proposed framework, and suggesting potential directions for future research.

## Literature review

Today, society’s dependency on the Internet and its associated service extends across various sectors, transforming into a vital infrastructure underpinning modern life. The growing reliance on digital technologies highlights the indispensable role of cybersecurity, a field that extends its influence across numerous disciplines. As digital systems become integral to everyday life and business processes, cybersecurity has evolved from an optional safeguard to a critical foundation. It forms the cornerstone of protection for other sectors, ensuring data remains secure, accessible, and private^12^.

In cybersecurity, AI is pivotal in improving the transparency and interpretability of machine learning models. This enables security experts to analyze and identify vulnerabilities, threats, or adversarial attacks with greater precision, strengthening and fortifying cyber-defense systems. The literature on AI-driven cybersecurity for secure software coding reflects significant advancements and ongoing challenges in this domain. This section scrutinizes existing research, highlighting key contributions, methodologies, and gaps in the field. The review is structured around the following four main themes.

### AI applications in cybersecurity

AI has become a game-changer in cybersecurity by automating intelligence practices in a timely, accurate, and holistic manner to fight against threats. Some of the major application areas of AI in cybersecurity include threat detection and prediction, in which case an AI system sorts through enormous datasets to identify warning signs that may indicate a security risk. Machines use algorithms to analyze past data to predict future security weaknesses and enable organizations to avert system attacks^5^. Furthermore, AI-oriented Intrusion Detection Systems (IDS) enable monitoring of network traffic in real-time to detect unique anomalies and malicious security events; advanced methods such as deep learning and support vector machines (SVMs) further improve the accuracy of zero-day attack detections^13,14^. AI is critical in fighting phishing and social engineering defense via natural language processing (NLP) and machine learning (ML) methods by which message contents are evaluated, while URL and sender credibility are checked^15^. Moreover, AI-driven tools analyze malware in detail using static and dynamic approaches and provide high detection accuracy for malware types using the Convolutional Neural Network (CNN)^16^. As regards dedicated data monitoring focusing on endpoint security, AI-based endpoint detection and response (EDR) systems observe the operations of devices to block them from unauthorized access and potential harm, ensuring enhanced detection of advanced persistent threats (APTs)^17^. AI further automates incident response activities, drastically increasing the speed required to remediate possible threats. Security Orchestration Automation Response (SOAR) systems employ artificial intelligence through platforms to mitigate threats efficiently^18^. Additionally, AI enhances cryptographic protocols and optimizes algorithms to improve secure communication methods^19^. Lastly, AI technologies also aid organizations in vulnerability management by creating risk ratings for prioritized assessments, facilitating targeted remediation actions, and leveraging predictive models to anticipate potential exploits by cybercriminals^20^.

### Cybersecurity risks and vulnerabilities: impact on software coding

So, a compelling impact on cybersecurity risks alongside exploitable vulnerabilities may guide the software coding results of how a set of systems would usually defend their security architecture. Unresolved code vulnerabilities function as attack pathways that enable attackers to compromise data through unauthorized entry and disrupt operations. The susceptibility of systems arises from poor input validation alongside insufficient secure authentication methods because these vulnerabilities would allow attackers to conduct SQL injection and cross-site scripting^21^. Secure coding practices have become more urgent than ever because sophisticated threats, including zero-day vulnerabilities, continue to appear. Zero-day exploits attack unidentified system flaws while systems remain unprotected, so developers must maintain continuous vulnerability management programs^22^. Strong encryption methods are vital because insufficient encryption strategies and aging cryptographic standards create security risks that permit sensitive data to reach unauthorized parties^23^. Third-party library combinations with framework integration during development carry potential risks that can affect project performance according to developmental models^24^. Coded components contain active vulnerabilities and harmful code requirements, demonstrating the essential character of strict dependency administration^25^. Disclosure attacks on model integrity caused by artificial intelligence and machine learning models in software development require builders to establish effective defensive measures during development^26^. Secure software development requires multi-faceted solutions that combine standardized, secure coding practices, periodic security examinations, and developer training about emerging cyber risks. According to research, every phase of software development must incorporate cybersecurity elements so organizations can defend against potential risks and create more robust systems^27,28^.

### Secure software coding practices

Programming software with secure coding principles protects both software systems' integrity, confidentiality, and availability of information^29^. These practices work to stop exploitive attacks and protect sensitive data while building trust within digital systems^30^. Developers support security through the least privilege principle by enforcing user and system privileges restrictions to the required levels necessary for their work tasks^31^. An application that handles essential user data must limit access to this data only through authorized program processes to decrease data exposure to unauthorized breaches^17^. Another cornerstone of secure coding is input validation and sanitization^28^. The main entry point for attackers is through user-supplied inputs, especially when inadequately validated^32^. Developers must use parameterized queries, vigorous validation checks, and output-escaping measures to reduce risks^33^. Secure programming requires developers to separate input parameters from actual statements, so they should employ prepared statements instead of direct SQL query concatenation. Software deployment requires a double strategic approach combining Code reviews and static analysis to find flaws beforehand^34^. Multiple team members assessing code during peer reviews help organizations receive different viewpoints regarding quality and security standards compliance. The code analysis tools SonarQube and Veracode monitor security threats by warning developers about threats consisting of insecure cryptographic usage and buffer overflows^34^. Integrating computerized tools in the SDLC results in lower post-sectional vulnerability correction expenses than traditional post-deployment detection practices^35^.

Additionally, incorporating secure libraries and frameworks is crucial^36^. OpenSSL cryptographic interfaces and secure APIs for authentication exist as tested established frameworks that help developers avoid putting insecure code into their systems^23^. Developers are responsible for library updates because outdated dependencies can act as security vulnerabilities^29^. Finally, comprehensive logging and monitoring provide insights into application behavior and potential security incidents^37^. The practice of secure logging involves both data anonymization along with measures to guarantee tamperproof log file management^38^. The monitoring software Splunk and ELK Stack notify teams about atypical behavior, which helps them respond quickly to cybersecurity threats^15^. Organizations create resilient information software systems by integrating strong cybersecurity practices across the Systems Development Life Cycle framework. Integration of AI technology within these cybersecurity practices proceeds at a slow pace^39^. For example, studies by^4,9,40,41^ showed that automatic secure code analysis tools perform marginally due to their limited capacity to understand contextual situations, which causes recognition mistakes and incorrect results. AI-based reinforcement learning techniques can improve these tools' versatile functionality and precise performance^9^.

### Integration of AI frameworks with maturity models

Integrating AI frameworks with maturity models is pivotal in aligning AI adoption with organizational readiness and strategic growth. AI frameworks, encompassing tools, methodologies, and technologies for developing and deploying AI solutions, provide the technical foundation for automating processes, enhancing decision-making, and improving predictive capabilities. Professional tools evaluate how well organizations handle multiple dimensions like strategy and technology through defined stages of development. Combining AI frameworks with maturity models helps organizations move beyond user-dependent AI use and creates alignment that produces optimal results from technology adoption despite minimized risks. For example, the Capability Maturity Model Integration (CMMI)^42^ addresses distinct AI-related concerns, especially ethical aspects, along with data integrity and model coordination requirements^9,43^. AI frameworks can assist organizations in systematically discovering operational skill deficit areas while collaborating with established maturity models to guide technology investments and specify the deployment of suitable solutions based on the organization’s current readiness levels^44^. Organizations with essential maturity struggle with data silos and knowledge gaps^43^. However, AI solutions (e.g., TensorFlow or PyTorch) need to be implemented strategically^45^ and workforce preparation. Better matured organizations can unite artificial intelligence and sophisticated strategies like generative AI and independent systems for complicated decision-making and innovation^43^. The integration also helps to improve governance and compliance^46^. Explainability features and fairness mechanisms (e.g., AI Fairness 360 or LIME) that AI frameworks use to enforce some levels of rigor before products are put into production also benefit organizations through maturity models that help them inject such known-good criteria needed to satisfy regulators and society^43^. AI implementation achieves a better return on investment when the investment connections are directly tied to measurable organizational business results. McKinsey conducted broad-scale research^47^ indicating that applying both AI structures and maturity models allows enterprises to deploy AI initiatives quickly and even quicker than with standalone AI tools. Results show that organizations following this combination are achieving 30% higher efficiency in adopting AI projects than their strategic alignment fellows the intersection of AI frameworks and maturity models presents organizations with a system that not only progresses transformational AI adoption but also integrates sustainability and scalability into the journey. Hence, integrating AI frameworks with organizational readiness assessments are advancing AI implementations by building ethical ways of attaining meaningful outcomes across various sectors.

With the recent advancement of Artificial Intelligence (AI) and Large Language Models (LLMs), AI-based code generation tools become a practical solution for software development^48^. GitHub Copilot, the AI pair programmer, utilizes machine-learning models trained on a large corpus of code snippets to generate code suggestions using natural language processing^48^. Large Language Models (LLMs) are a type of natural language processing (NLP) technique based on deep learning that is capable of automatically learning the grammar, semantics, and pragmatics of language and generating a wide variety of contents^49^. Due to the extensive number of parameters and large-scale training datasets, LLMs have demonstrated powerful capabilities in NLP, often approaching or even surpassing human-level performance in NLP tasks such as text translation^50^ and sentiment analysis^51^. Recently, AI code generation tools driven by LLMs that have been trained on large amounts of code snippets are increasingly in the spotlight (e.g., AI-augmented development in Gartner Trends 2024^52^. AI tools can produce solutions that outperform those created by novice programmers in the case of simple and moderately complex coding problems^53^. Generative artificial intelligence (GenAI) technologies using LLMs (large language models), such as ChatGPT and GitHub Copilot, with the ability to create code, have the potential to change the software-development landscape^54^.

However, in this regard, the proposed “AI-driven Cybersecurity Framework for Software Development Based on the ANN-ISM Paradigm” outsmarts the traditional cybersecurity maturity models, such as the NIST framework or CMMI, because of its advanced nature of using predictive AI methods on continuous learning with the ISM. Therefore, here are some reasons why this model beats this already existing framework:

- *Proactive threat detection and prevention*: The main strength of the ANN-ISM paradigm is the predictive capacity with Artificial Neural Networks (ANN). With every new piece of historical or real-time data, ANN learns and predicts future possible threats before the situation starts to create trouble. As it identifies emerging cybersecurity threats proactively, it has the advantage of early intervention and prevention over traditional models.
  The NIST Cybersecurity Framework and CMMI (Capability Maturity Model Integration) are excellent in risk management, compliance, and process improvement, but what is reactive? Framework, on the other hand, stresses structured practices for dealing with cybersecurity risks and vulnerabilities. Still, they lack real-time predictive capabilities for preemptively mitigating the threats involved, unknown and fluid.
- *Adaptability to emerging threats*: In the ANN-ISM Paradigm, the ANN component of your model allows continuous learning. This system adapts to the emerging signals of new attacks as new data is being processed and evolves with the pace of cyber threats. Integrating the ISM framework further refines the security policies to make the system more flexible and adaptive to dynamic environments.
  The frameworks of the NIST and CMMI approach are static. Although they deliver sound recommendations for the content of improved cybersecurity practices in the future, they cannot automatically react to new kinds of threats or modifications to the environment without human supervision. For example, while NIST relies on a risk-based management approach and its best practices, it dictates continuous human oversight to change controls and protocols to newly acquired threat intelligence.
- *Real-time threat detection and response*: The same cybersecurity incident detected by our model is also detected in real-time, and our model reacts to it automatically, taking needed actions immediately. Hence, the framework combines AI threatened detection with ISM to generate real-time alerts and countermeasures.
  NIST and CMMI frameworks support the create security processes in detail but not for real-time threat detection and response. Though NIST focuses on continuous improvement through monitoring, it does not provide the ability to act immediately when confronted with threats, such as the ANN-ISM model. In the same light, CMMI tailors more towards process maturity and organizational capability than on-the-spot cybersecurity incident management.
- Scalability and Flexibility: Integrating neural networks with ISM in your framework makes it highly scalable (ANN-ISM Paradigm). It scales to support an ever-growing volume of data and to meet an ever-increasing security demand over time without negatively affecting its performance. Machine learning is employed within the framework to improve the system and better identify complex and unlearned threats with the scale of data.
  Both NIST and CMMI frameworks are excellent means of providing a structured approach to the idea of cybersecurity maturity. However, they are generally less flexible when solving new, complex, or growing problems. These models can help set a baseline and improve the security process. However, they do not inherently scale well to account for increasing threat, complexity, and data without a manual adjustment or update.
- *It facilitates the management and continuous improvement of automated process*: The ISM component of the model integrates into the framework a constant improvement cycle known as the ANN-ISM Paradigm. The system uses machine learning and security management processes, evaluating its processes constantly and reacting to that evaluation by adjusting its processes; therefore, the system can continuously improve its cybersecurity procedures via new data and shifts in the threat landscape.
  As for the CMMI Framework, it focuses on the organizational processes and maturity of capabilities and is more concerned with continuous process improvement. In itself, it does not incorporate that learning from data is automated. The NIST framework gives guidelines for better security postures. Still, it is dependent on periodic manual updates and does not have an automatic upgrade process that allows continuous improvement of security posture continuously.
- *Cost-effectiveness over time*: The initial investment for the ANN-ISM model can be higher because it couples with AI and continuous learning capability. However, this gets less expensive over time by eliminating manual intervention, increasing the security posture, and decreasing incident response costs. In that, it excludes the expense of preventing the attacks before they happen, making the cost of cybersecurity management low.
  Frameworks such as NIST and CMMI frameworks involve more significant operational costs, employing manual processes, consistent updates, and regular monitoring and aligning the process toward improvement. While these are valuable processes for handling cybersecurity, they are not by themselves threats detection automation or continuously evolving without human help.
- *Holistic security approach*: The ANN-ISM paradigm provides a holistic view of cybersecurity where AI-based predictive analytics is coupled with structured security management. This ensures that all parts of cybersecurity, from threat detection to policy management, work harmoniously to give a holistic solution that targets the technical and managerial parts of cybersecurity.
  While both the NIST Cybersecurity framework and CMMI Frameworks supply beneficial guidelines for its organization to bolster its cybersecurity methods, they are more scattered in their stance. NIST deals with risk management and setting up controls, and CMMI covers the process maturity to a large extent without fully incorporating predictive technology (CTIS) and continuous learning of security infrastructure.

Unlike the traditional models of NIST and CMMI, the Cybersecurity Framework for Software Development Based on the ANN—ANN-ISM paradigm provides a more dynamic, adaptive, and predictive solution. NIST and CMMI frameworks do provide a good framework to manage and improve security practices over time; however, they are reactive and costly to tackle new and unanticipated threats, as they require manual intervention. The ANN-ISM paradigm powered by AI makes a more real-time, more automated, and easier to scale to changing environment cybersecurity problem with the trade-off that many of the weaker tools of human bureaucracy are lost.

## Research methodology

**Fig. 1:**
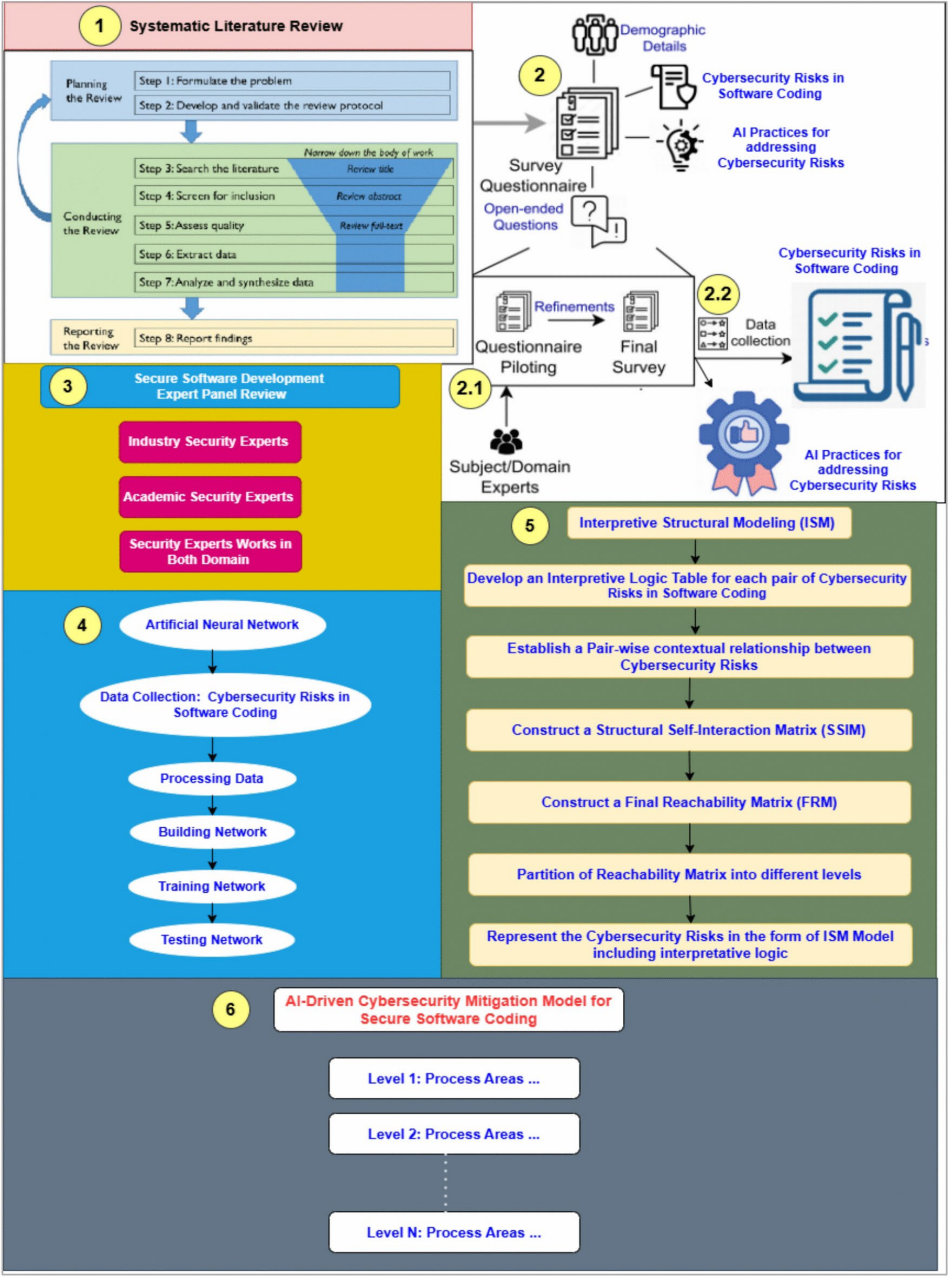
Research flow framework.

The research methodology for this study is designed to ensure systematic and rigorous processes required for constructing a solid security mitigation model. This methodology is structured into five key phases: Systematic Literature Review, Questionnaire Survey, Expert Panel Review, Artificial Neural Networks Analysis, and Interpretive Structural Modeling (ISM). Each phase contributes to an iterative process that assists in achieving the overarching goal of developing a combined AI-Driven Cybersecurity Mitigation Framework for Secure Software Coding: An ANN-ISM Framework. Figure 1 presents the graphical research framework for this study.

### Phase 1: systematic literature review (SLR)

This study aimed to produce a holistic view of the scientific literature on secure software coding to understand the cybersecurity risks and practices available to address these risks. It offers valuable insights from both researchers and practitioners. Researchers seek a comprehensive source of information that provides an overarching perspective on the latest advancements in secure software development. On the other hand, practitioners aim to understand emerging trends and technologies from the research community that could be effectively applied in real-world scenarios. Established guidelines were implemented using an SLR^28,55–57^. Under the guidance of the co-authors, the first author developed a thorough review protocol and performed in-depth searches, screened the studies, and executed data extraction under the supervision of the second author, who has extensive experience with SLRs. All the authors equally contributed to the data analysis process. The first author organized the data into tables and graphs and drafted the analysis report, while the remaining authors provided feedback throughout, offering notes and participating in discussions.

#### Research questions

This research aimed to answer various research questions to comprehensively understand cybersecurity risks associated with software coding and the corresponding AI practices. The research questions formulated in this study are intended to deepen the understanding of these critical issues:

##### RQ1

 What are the potential vulnerabilities and cybersecurity risks identified through a literature review and in real-world industry experiences that need to be addressed for secure software coding?

##### RQ2

 What are the best AI practices to address cybersecurity risks to software development organizations in secure software coding, as identified through literature review and real-world industry?

##### RQ3

 How can a robust AI cybersecurity mitigation model be designed with the help of ANN-ISM methods to improve secure software coding?

#### Search strategy

According to the guidelines^55,58–60^, only the population and intervention variables are used to perform the systematic study. SLR is a preliminary study giving an overview of the status of research in a field^21,24,56^. SLRs are traditionally focused on illuminating gaps in the current state of knowledge and providing an overview of topics, methods, and outcomes of the literature within a specific domain^3,59,61^. It is more activities around the identification of the literature and its categorization than the comparison of outcomes or definitive conclusions. To narrow down the area of the research, we established these two variables (population and intervention) as the core keywords to develop search strings for this study. In software engineering, “population (P)” can refer to subgroups like specific roles within the industry, types of software engineering, or application domains. Including these groups may be necessary to the research due to challenges specific to these groups or certain characteristics^27^. For this particular study, the focus was on research papers related to secure software coding.

The term “*intervention* (I)” in software engineering typically refers to any technique, tool, technology, or process used in the development, maintenance, or improvement of software systems^58^. In the review, we chose not to focus on any specific intervention related to secure software coding. Instead, we aimed to explore various interventions to gain a broad understanding of the current research landscape and identify potential cybersecurity risks and AI mitigation practices within the field. Based on the population, intervention, and research questions, we developed the following research string:

((“Software Coding” OR “Secure Software Development” OR “Software Implementation” AND (“Cybersecurity Risks” OR “Cybersecurity Issues” OR “AI Practices” OR “Artificial Intelligence Models” OR “Artificial Intelligence Tools” OR “Artificial Intelligence Frameworks” OR “Artificial Intelligence Approaches” OR “Artificial Intelligence Techniques” OR “Artificial Intelligence Standards”)).

The guidelines^55,58–60^ recommend using IEEE, ACM, ScienceDirect, SpringerLink, Wiley Online Library, and Google Scholar indexing databases for SLR. We applied the search queries across the mentioned databases, examining all available fields. Table 1 shows the number of studies retrieved from each source.

**Table 1:** Number of studies obtained in each database.

Digital libraries	Search string findings	Initial selection	Final selection
IEEE Xplore	98	28	19
ScienceDirect	150	33	18
ACM	135	39	15
Wiley Online Library	60	10	8
SpringerLink	230	50	16
Google Scholar	2021	60	14
Total	2694	220	90

#### Study filtration and selection

In this phase, the collected papers were manually filtered based on the inclusion and exclusion criteria outlined in Table 2. These criteria were derived from the research questions. To maintain objectivity, we only included accessible peer-reviewed papers that were published in conferences, workshops, or journals. The paper selection is based on the title, abstract, and, when necessary, full-text review. A think-aloud approach describes one study's inclusion and exclusion process to ensure clarity and consensus. Each paper’s title and abstract are thoroughly examined during the selection process. Papers with titles indicating secondary studies were included. If a title explicitly referenced “Secure Software Coding”, the first author thoroughly examined the abstract. If the abstract addresses secure software coding, the paper will advance to the next stage. For titles without this direct reference, the abstract was reviewed for relevance. If ambiguity remained, the paper was skimmed for key information, such as objectives, methodology, results, and conclusions, to ascertain its relevance to secure software coding. The other authors validated the selection process by randomly reviewing papers using the same procedure. An agreement was reached after discussions, and the inclusion/exclusion criteria were updated accordingly. The final criteria were then applied to all studies. Papers that meet at least one of the exclusion criteria in Table 2 were eliminated. Conversely, papers that have not been excluded and meet at least one of the inclusion criteria were retained for the review.

**Table 2:** Study selection criteria applied in the study selection phase.

Study selection criteria	
Inclusion criteria (IC)	IC1: Articles must be published between the years 2010 and 2025 IC2: The article must be peer-reviewed and published in a Journal, conference, or workshop IC3: The article must be accessible online IC4: Article discussing some AI approaches, models, algorithms, considerations, challenges, risks, practices, and frameworks for cybersecurity in software coding IC5: Articles must be in English
Exclusion criteria (EC)	EC1: Article not peer-reviewed EC2: The article is not a full research paper EC3: Article not in English EC4: Articles proposing AI approach, model, algorithm, consideration, challenge, risk, practice, and framework for cybersecurity in software coding EC5: The non-reputable publisher's articles

#### Study quality assessment

Petersen et al.^58^ suggest performing a quality assessment without setting strict criteria to avoid excluding potentially relevant systematic review resources. Therefore, we devise the following quality assessment approach (Table 3).

**Table 3:** Study quality assessment criteria.

Quality assessment criteria (QAC)	Description
QAC1: Relevance of the research problem	Does the paper clearly state the relevance and importance of AI and cybersecurity in software coding?
QAC2: Literature review quality	Are the key studies, trends, and gaps in the field adequately defined?
QAC3: Methodological rigor	Are the methodologies used to generalize AI and cybersecurity in software coding well-explained, appropriate, and reproducible?
QAC4: Clarity and accuracy of the results	Are the results of AI effectiveness in secure software coding presented clearly with tables, graphs, or other visual aids?
QAC5: Contribution to the field	Does the paper contribute new insights, models, or techniques for applying AI mitigation for cybersecurity in software coding?
QAC6: Presentation and writing quality	Is the paper well-structured, logically organized, and easy to follow?

#### Data extraction

Following a comprehensive review of each primary study, we gathered data directly from the paper. This included qualitative and quantitative information: qualitative data comprised (i) Cybersecurity Risks in Software Coding and (ii) AI practices for addressing cybersecurity risks. The quantitative data included the sample size or the number of participants, data sources, and the length of datasets.

#### Data synthesis

This SLR synthesized data based on themes, including cybersecurity risks and AI mitigation practices. Thus, the synthesis encompassed the most prevalent cybersecurity risks in software coding, Insecure Coding Practices, Vulnerable Dependencies, Poor Error Handling, Weak Authentication/Authorization, Misconfigured Security Controls, Inadequate Encryption, Cross-Site Scripting (XSS), Insufficient Logging and Monitoring, Race Conditions, Inadequate Security Testing, and Supply Chain Attacks. Thus, by outlining a clear map of the development of AI security practices in software coding, the paper offers an extensive study of the current trends and achievements in the field as well as the directions that require further research to prevent potential barriers to the successful implementation of secure software coding practices techniques.

#### The findings

The detailed report of the SLR results is prepared and structured under section “Results and discussions” of this paper. Therefore, through these simple and structured steps, the literature review will comprehensively identify cybersecurity threats and the corresponding AI-driven mitigation methods in software coding.

### Phase 2: questionnaire survey

We report the results of an empirical study conducted by means of an online survey from which input was obtained from experts and practitioners in secure software development. They discussed their experiences in cybersecurity risk management and how their practices shaped software coding.' The use of an online survey for this study provided various benefits^62^. Most importantly, it removed the need for meeting protocols or international travel, making it possible to gather data efficiently across continents. Moreover, using affordable and convenient online resources such as Google Online Form made the implementation process practical. Through a series of sampling phases, the survey was designed. The analysis and synthesis of the design involved the consideration of several systematic and unsystematic sampling methods in the development of the sample questions^63^. Given the survey type, an online survey was chosen as a non-scientific data gathering and extraction mechanism^46,64,65^, as it proves to be more practical than collecting data directly from experts situated in different countries.

#### The pilot of questionnaire survey

A pilot study consisted of experts from secure software development organizations to assess the questionnaire survey as such experts include “Cyber-Physical Systems Research Group, the University of Southampton”, “College of Computer and Information Sciences, King Saud University", “Software Engineering Research Group (SERG_UOM) Pakistan.” The questionnaire survey is then revised based on expert feedback.

#### Data collection sources

Expert data was collected using snowball sampling^66^. Contact was made through e-mail and many social media sites, including ResearchGate, Facebook, LinkedIn, and Gmail. (Out of 75 responses by secure software development organization survey participants, 10 were discarded as they did not have a cybersecurity focus. The resulting 65 survey responses of the final dataset were manually reviewed and were used for subsequent statistical analysis. The breakdown of respondents included a higher percentage of those holding Master's degrees, whilst fewer of those with PhDs. Most respondents were from major network security companies, and many held decision-making positions.

**Fig. 2:**
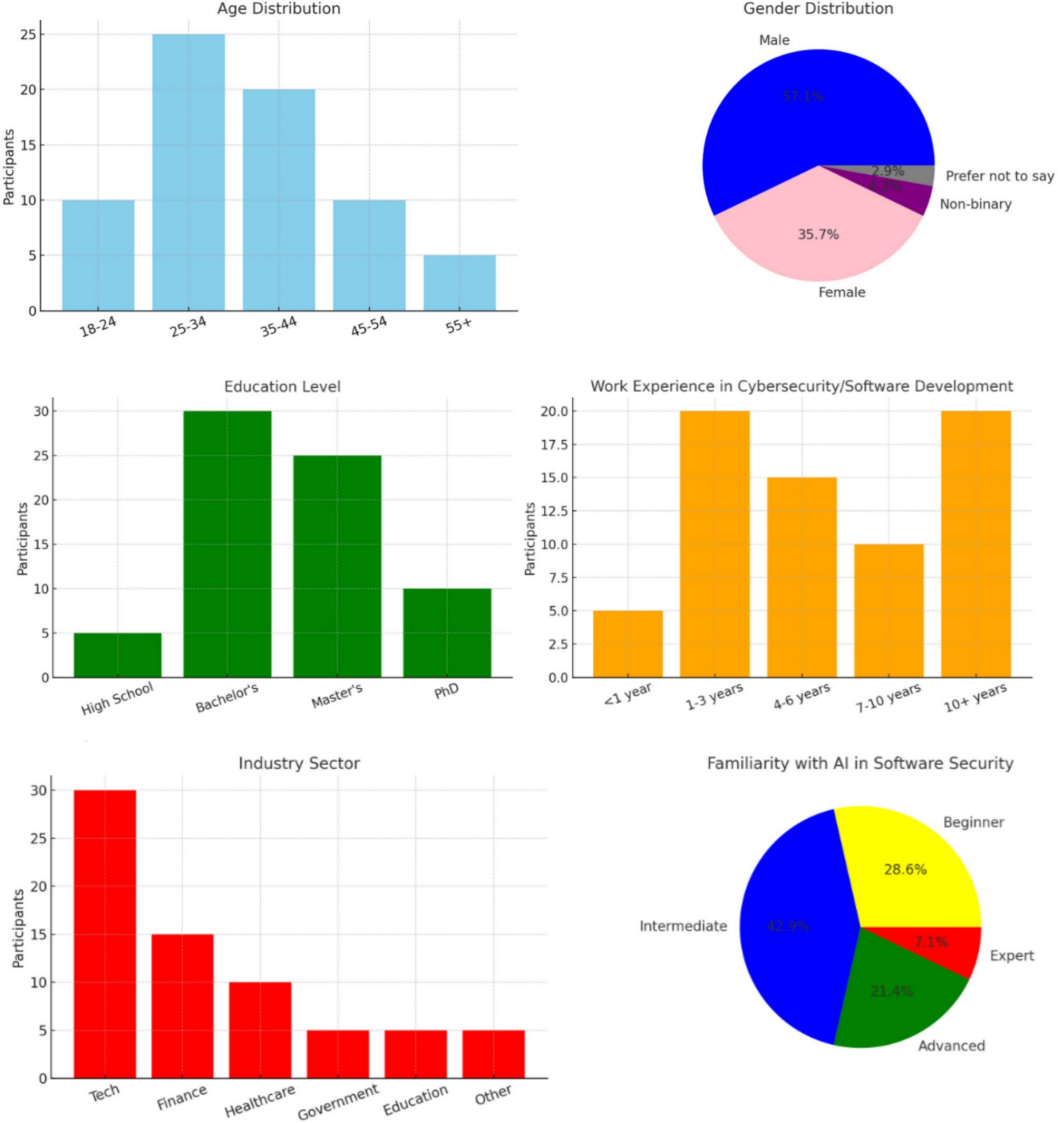
Demographic details of survey participants.

Figure 2 presents the demographic details of the 70 survey participants for identifying cybersecurity risks and practices that impact secure software coding. We designed survey questions (SQs) to collect data from the survey participants, as presented in the Appendix.

### Phase 3: expert panel review

To ensure the credibility of the research, the Expert Panel Review is carried out to confirm the moderation of the SLR and the Questionnaire Survey results and improve the research methodology. It involves a panel of software developers, cybersecurity experts, artificial intelligent, and security systems modeling specialists.

The authors are chosen with an emphasis on their previous work and impact on software coding, AI, and advanced modeling. It consists of academic and professional experts, and some progressive thinkers are also a part of this panel.

#### Conducting the review

It is a structured process, and the expert panel is assembled using rounds of Delphi workshops. In these sessions, the experts discuss the outcome of the SLR and survey studies and offer their opinions on the research process and possible enhancements.

#### Incorporating feedback

The communication with the expert panel is conducted in a structuralized manner to integrate the feedback into the research process actively. This involves refining the study questions, modifying the areas of focus in the two models of ANN and ISM, and establishing that the study covers all the significant cybersecurity risks and AI mitigation practices in secure software coding.

Seventeen cybersecurity specialists were selected based on their professional expertise in cybersecurity risks and practices for securing software coding. They represent three key groups: security industry practitioners, academic researchers, and experts with experience in both domains. These experts evaluated the significance of various cybersecurity risks in software coding using a structured rating scale, where risks considered less than 10% important received a value of 1. In comparison, those rated between 90–100% importance were assigned a value of 10, with intermediate values increasing in 10% increments.

As highlighted in Table 4, the evaluations made by the experts were used to derive the pair-wise matrix relationships between the cybersecurity risks in software coding. These evaluations were further analyzed using the ANN and Interpretive Structural Modeling (ISM) methods.

**Table 4:** Potential vulnerabilities and cybersecurity risks identified through literature review and real-world industry.

Code #	Cybersecurity risks in software coding (CRSC)	Impact percentage (Literature) (%)	Impact percentage (Industry Survey) (%)
CRSC1	Insecure coding practices	25	26
CRSC2	Vulnerable dependencies	30	18
CRSC3	Poor error handling	20	15
CRSC4	Weak authentication/authorization	15	12
CRSC5	Misconfigured security controls	12	10
CRSC6	Inadequate encryption	10	8
CRSC7	Cross-site scripting (XSS)	8	7
CRSC8	Insufficient logging and monitoring	8	6
CRSC9	Race conditions	6	5
CRSC10	Inadequate security testing	12	5
CRSC11	Supply chain attacks	8	4
CRSC12	Insider threats	6	4
CRSC13	Insecure APIs	15	3
CRSC14	Malware in codebase	4	2
CRSC15	Compromised CI/CD pipelines	3	2

### Phase 4: artificial neural network (ANN)

In the fourth phase of this study, we utilized the ANN approach. ANN is particularly suitable for analysis because it can process input data without requiring a specific equation format. Moreover, it can be easily adapted to handle new data sets. One of its key advantages is its ability to manage incomplete or insufficient data inputs^67^. Compared to methods such as sequential equation modeling, multiple linear regression, MDA, and binary logistic regression, ANN generally produces more accurate predictions. Interpretive structural modeling (ISM) is commonly used to analyze how predictors influence a dependent variable. Still, traditional linear methods like ISM may oversimplify human decision-making processes by only capturing linear relationships^68,69^. ANN, a widely recognized and essential AI model, overcomes this limitation by simulating decision-making through its ability to map nonlinear relationships, according to Leong et al.^68^. ANN multi-layer perceptual form is designed to capture complex input/output mappings, similar to how the human brain functions. A significant strength of ANN models is their ability to identify nonlinear and non-compensatory relationships^70^.

In summary, ANN models outperform traditional linear methods in predictive accuracy and exhibit remarkable flexibility and robustness^64^. However, ANN is unsuitable for investigating causal relationships or hypothesis testing^68,71^. To address this, a two-step method combining ISM and ANN was developed.

#### ANN training

Training an ANN involves modeling the implicit input/output relationships by adjusting internal weights. The provided input/output pairs take the form:

1 $$ {\text{S }} = \, \left( {{\text{d1}},{\text{ x1}}} \right), \, \left( {{\text{d2}},{\text{ x2}}} \right), \, ...., \, \left( {{\text{dNi}},{\text{ xNi}}} \right) $$

where xi is a random sample of input parameters, and di is a random sample of corresponding responses. These datasets implicitly reveal nonlinear connections between inputs and outcomes. The objective is to create an ANN model capable of automatically learning this hidden task. Typically, the output of an ANN is expressed like w_ij_y_i_ + b_i_

2 $$ {\text{y }} = {\text{ y}}\left( {{\text{x}},{\text{ w}}} \right) $$

where x represents the input parameters, y represents the ANN’s output, and w is the vector of unknown weights. Solving an optimization problem identifies the appropriate weights. This process minimizes the deviation between the model’s predicted output and target values. The optimization problem is expressed as:

3 $$ {\text{w}} * = {\text{ min x ET }} = {\text{ min x i }}\left| {\left| {{\text{ di }} - \, \gamma \, \left( {{\text{xi}},{\text{ w}}} \right) \, } \right|} \right| $$

where ET represents the sample standard error. Various methods exist to solve this optimization problem, with back propagation, introduced by Hertz et al.^72^, the most widely used. In this method, weights are updated using the estimated gradient of the error function concerning the weights, leading to more accurate predictions:

4 $$ \upomega_{{{\text{next}}}} = \, \omega_{{{\text{now}}}} - \, \eta \, \alpha {\text{E}}\tau / \, \alpha \upomega $$

Hertz et al.^72^ define the learning rate as h. The initial weights are assigned randomly, and the process continues until the optimization in Eq. (3) is satisfied. The function is optimized by recalibrating the weights and biases, aiming to minimize the mean square error until the model achieves the desired accuracy.

Wi and bi(s) optimize mean square error. This procedure will continue until the desired level of precision is reached. Alnaizy et al.^73^ describe the calibration as follows:

5 $$\text{Vi }= \sum_{i=1}^{n}\text{wijyi }+\text{ bi}$$

where the bias bi adjusts the sum of the inputs and weights. A transfer function, also called an activation function, is applied to transform the sum Vi. This transformation produces the:

6 $$ {\text{Zi }} = {\text{ f }}\left( {{\text{Vi}}} \right) $$

#### Performance of ANN training

The ANN’s performance is assessed using metrics like Root Mean Squared Error (RMSE), R-squared (R^2^), and Average Absolute deviation (AAD), calculated as follows:

7 $$ {\text{RMSE }} = { }\left[ {\frac{1}{n}\mathop \sum \limits_{i = 1}^{n} \left( {Yi - Yid} \right)^{2} } \right]^{0.5} $$

8 $${R}^{2}=1-\frac{{\sum }_{i=1}^{n}{(Yi-Yid)}^{2}}{{\sum }_{i=1}^{n}{(Yi-Yin)}^{2}}$$

9 $$ AAD = \left[ {\frac{1}{n}\mathop \sum \limits_{i = 1}^{n} \frac{{\left( {Yi - Yid} \right)}}{Yid}} \right] \times 100 $$

Here, Yid is the observed data, Yi is the predicted data, Ym is the median of the observed data, and n is the number of data.

### Phase 5: interpretive structural modeling (ISM) analysis approach

**Fig. 3:**
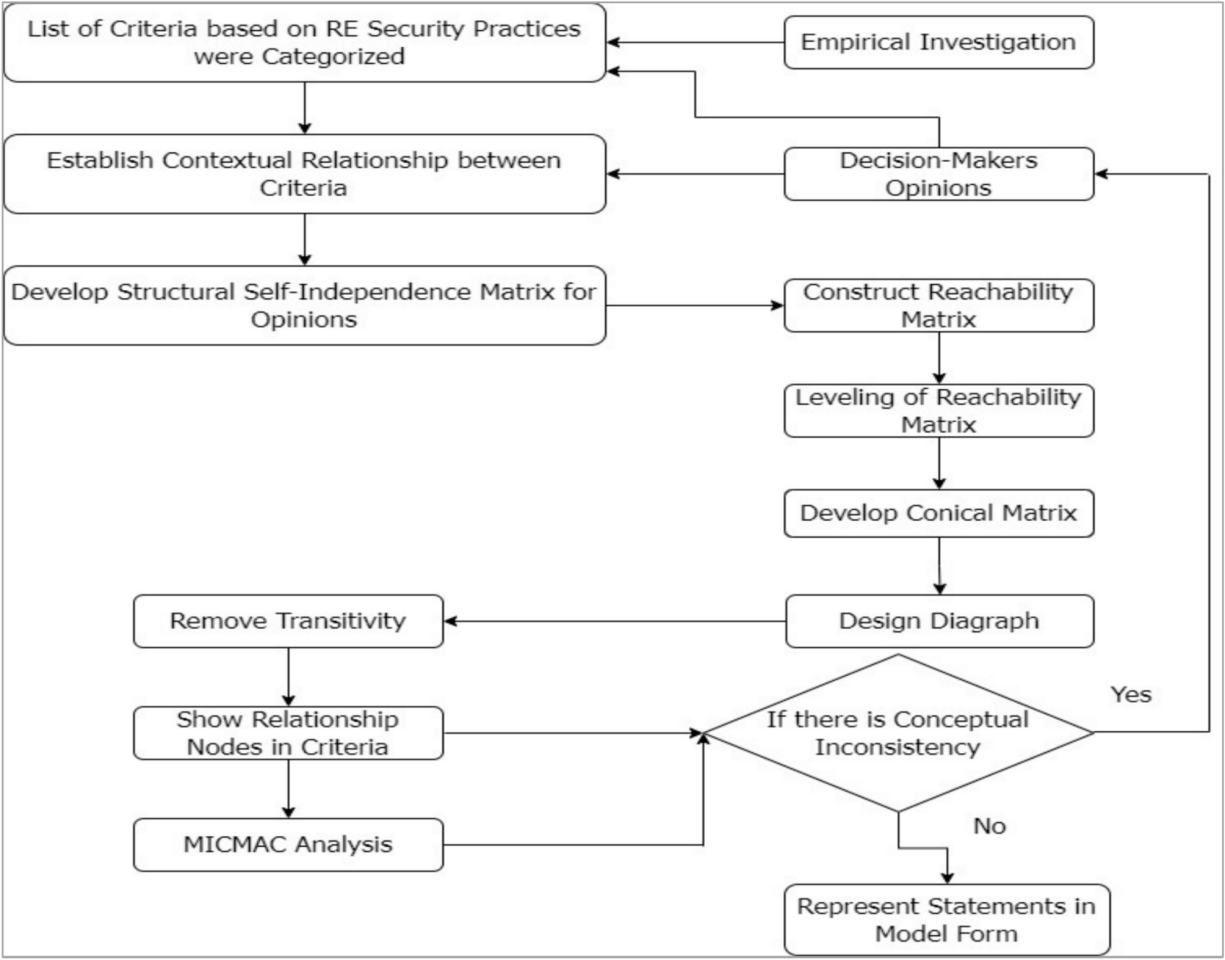
ISM approach.

The fifth step of this study used the ISM method to classify and determine the relationships of cybersecurity risks in software coding. In^74^, ISM was proposed to synthesize complex associations between systems and the elements composing the system. Competence building is done by establishing order and direction on various components to build a comprehensive model hierarchically. ISM methods can also be used because they capture these relationships well through graphic representation and organized language^75^. ISM is beneficial for obtaining relationships between many variables when more complex connections are concerned^76–78^. Several other researchers have employed this approach to build a more nuanced understanding of the systems they are investigating^79–86^. Figure 3 depicts the ISM approach to map and classify software coding security threats.

### Phase 6: development of an AI-driven cybersecurity mitigation for secure software coding: an ANN-ISM framework

In the final phase of this study, we merge the output data of ANN & ISM to create the proposed AI-driven cybersecurity Mitigation Model for Secure Software Coding. We refer to section “Development of ai-driven cybersecurity mitigation model for secure software coding: using ANN-ISM approach” for more detail.

## Results and discussions

In this section, we summarized the results of the hybrid research approach through SLR, empirical survey, expert panel review, ANN, and ISM. The answers to the research questions raised in section “Research methodology” are provided in the subsequent subsections.

### Potential cybersecurity risks and vulnerabilities in software coding

Literature review and industry applicability concerning the vulnerabilities and security threats in the soft-ware coding practices If these vulnerabilities in their software go unaddressed, it can result in serious security risks, financial losses, and reputational damage. Looking at vulnerabilities from the intersection of academic research and real-world use shows us the theory whilst also demonstrating the reality. Such literature reviews encourage insights and paradigm developments whilst also revisiting previously studied case studies, which together can serve to inform better inform foundational insights about software vulnerabilities better inform foundational insights about software vulnerabilities better. In contrast, industry surveys reflect the current threat landscape, practical implications, and actionable data, which are based on real-world cases and connect research with practice. This two-pronged approach ensures that both software developers and cyber-security professionals, as well as those who help formulate government policy, understand the theoretical risks involved and are given actionable solutions that are cognizant of the threats posed by the modern threat landscape.

Additionally, this breaking down of vulnerabilities allows us to focus on significant problems such as insecure coding practices, weak authentication mechanisms, and poor encryption, which can lead to focused action. Organizations can develop software more securely by identifying these risks early and taking steps to mitigate them. Organizations increase the robustness of their software systems but also remain compliant with regulatory compliance and build end-user trust in an increasingly dynamic digital environment. Table 4 presents potential vulnerabilities and cybersecurity risks identified in the literature review and real-world industry.

Table 4 presents that the most cited security risk in software coding is “insecure coding practices”. The implementation flaws arising from insecure coding practices allow attackers to exploit the application to perform adverse actions, leading to undesirable consequences^87^. These flaws can be classified as injection and logic flaws. Some other cited cybersecurity risks and vulnerabilities can also impact secure software coding. Cybersecurity vulnerabilities significantly impact software coding by exposing systems to potential breaches, compromising data integrity, and undermining the reliability and trustworthiness of the software^88^. Poor error handling can reveal sensitive information or system vulnerabilities to attackers^89^. At the same time, weak authentication or authorization mechanisms can lead to cybersecurity risks in software coding by allowing unauthorized access to sensitive data and critical system functions^90^. Misconfigured security controls create cybersecurity risks in software coding by exposing vulnerabilities that attackers can exploit to gain unauthorized access or disrupt system functionality^91^. Inadequate encryption in software coding compromises data confidentiality and integrity, leaving sensitive information vulnerable to unauthorized access and exploitation^92^. Cross-site scripting (XSS) remains a threat due to software coding vulnerabilities that enable attackers to insert nefarious scripts into web applications, resulting in user data being compromised, sensitive information being stolen, and undermining the integrity of applications^93^. In software coding, insufficient logging and monitoring diminishes the capability to detect, investigate, and respond to a security incident, allowing the systems to be susceptible to undetected breaches and protracted exploitation^94^. Race conditions are also highly influential in software coding as they lead to unpredictable behavior and bugs when multiple threads or processes read and write shared data concurrently^34^. Inadequate security testing may result in gaps in security testing, leading to higher chances of it being exploited, which may hinder the application’s integrity and user data^35^. Supply chain attacks compromise the integrity of software coding by injecting malicious code into dependencies, tools, or updates used during development^95^. Insider threats can compromise software coding by introducing malicious code, exploiting vulnerabilities, or leaking sensitive project information, jeopardizing security and integrity^96^. Insecure APIs can introduce significant cybersecurity risks in software coding by exposing vulnerabilities that attackers can exploit to access sensitive data or compromise system integrity^97^. Malware embedded in the codebase can pose significant cybersecurity risks, compromising software integrity, exposing sensitive data, and creating vulnerabilities for potential exploitation^16^. Compromise CI/CD pipelines can introduce significant cybersecurity risks in software coding by enabling unauthorized access, injecting malicious code, or disrupting secure development workflows^98^.

**Fig. 4:**
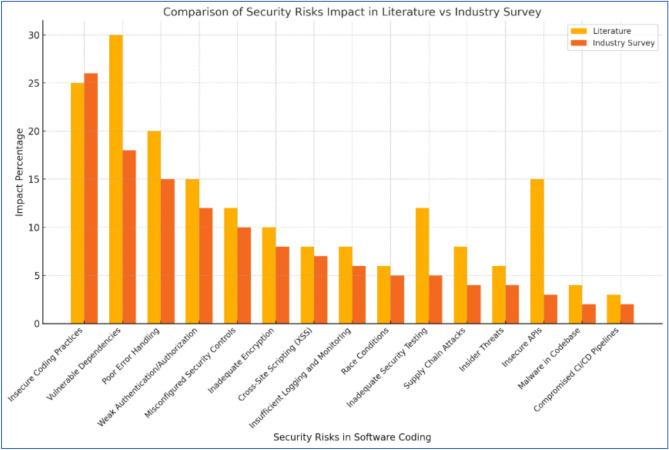
Comparison of software security coding risks as identified through literature review and surveys.

**Fig. 5:**
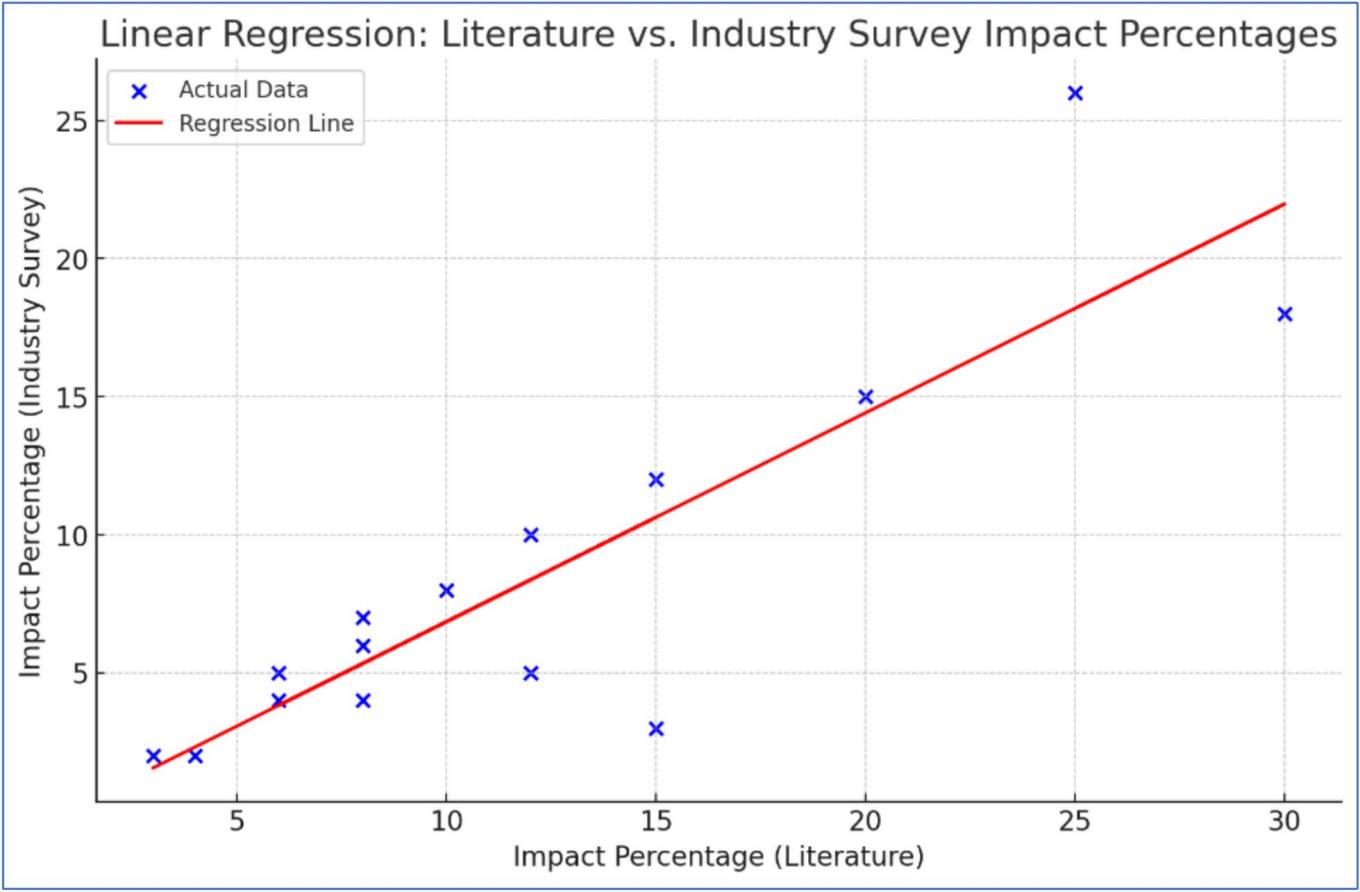
Linear regression of software security coding risks as identified through literature review and surveys.

Figures 4 and 5 represent the comparison and linear regression of software security coding risks identified in literature and surveys. We performed a statistical correlation measure to describe the extent to which the literature review and survey findings (software security coding risks) are related. This helps us understand whether and how strongly pairs of variables are associated. The correlation coefficient is a value that ranges between − 1 and 1. In correlation measure, + 1 indicates a perfect positive correlation, − 1 indicates a perfect negative correlation, and 0 indicates no correlation. The Pearson correlation coefficient, which is the most common type of correlation, is calculated using the following formula:

10 $$r= \frac{\sum ({x}_{i}-\overline{x })({y}_{i}-\overline{y })}{\sqrt{{\sum ({x}_{i}-\overline{x })}^{2}{\sum ({y}_{i}-\overline{y })}^{2}}}$$

where ${x}_{i}$ and ${y}_{i}$​ represent individual values of the two variables (Literature Review and Industry Survey), and $\overline{x }$ and $\overline{y }$​ are their respective means. The mean impact percentage of literature is 12.13, while that of the industry survey is 8.47. The sum of the product of deviations is 638.07, with standard deviations of 29.05 for the literature and 25.33 for the industry survey. Substituting these values, the Pearson correlation coefficient is $r= \frac{638.07}{29.05 \times 25.33}$ ≈ 0.867.

This result indicates a strong positive linear relationship between literature review findings and industry survey results, suggesting significant alignment between academic research and real-world cybersecurity risks.

### Best AI practices for addressing potential cybersecurity risks and vulnerabilities in software coding

AI significantly contributes to detecting and preventing cybersecurity threats in secure software coding by leveraging its ability to analyze vast amounts of data, identify patterns, and predict potential vulnerabilities. A detailed exploration of its contributions, supported by a literature review and real-world applications, includes the following aspects in Table 5.

**Table 5:** AI practices for addressing cybersecurity risks in software coding.

S. No	AI practices for CRSC 1: insecure coding practices	Literature review impact (%)	Real-world survey impact (%)
1	Automated code analysis tools	85	75
2	Secure coding standards enforcements	72	82
3	Threat modeling and risk assessment	78	65
4	AI-powered code reviews	75	72
5	Secure framework recommendations	60	70
6	Training and education	68	55
7	Monitoring and logging automation	65	58
8	CI/CD pipeline security integration	60	55
9	Real-time vulnerability patching	50	58
10	Phishing and social engineering AI	50	65
AI practices for CRSC 2: vulnerable dependencies			
11	Automated dependency scanning	80	79
12	Version control and dependency updates	70	70
13	Risk prioritization using AI models	65	75
14	Natural language processing for vulnerability analysis	60	65
15	Machine learning for vulnerability prediction	68	78
16	Dependency mapping and visualization	82	72
17	Secure coding guidelines recommendation	63	72
18	Continuous integration and continuous deployment (CI/CD)	88	80
19	Dynamic vulnerability testing	76	66
20	AI-enhanced software composition analysis (SCA)	80	88
AI practices for CRSC 3: poor error handling			
21	Error classification models	82	80
22	Automated vulnerability scanning	90	85
23	Dynamic runtime monitoring	80	85
24	Predictive code analysis	83	89
25	Natural language processing (NLP) bots	70	65
26	AI-driven test case generation	92	88
27	Automated code review	79	87
28	Adaptive learning systems	78	82
29	Self-healing code frameworks	82	79
30	Error handling standards enforcement	84	86
AI practices for CRSC 4: weak authentication/authorization			
31	Enforce strong password policies	87	79
32	Implement multi-factor authentication	80	85
33	Role-based access control (RBAC)	80	65
34	Use secure token-based authentication	80	90
35	Input validation and sanitization	65	75
36	Implement session management controls	85	60
37	Adopt secure communication protocols	95	85
38	Regular security testing	70	80
39	Centralized identify management	65	60
40	Monitor and log authentication events	70	50
41	Continuous training for developers	60	70
AI practices for CRSC 5: misconfigured security controls			
42	Automated security scanning	79	80
43	Real-time code analysis	70	85
44	Intelligent configuration templates	75	60
45	Anomaly detection in code behavior	70	75
46	Continuous learning and updates	78	68
47	Role-based access recommendations	65	85
48	Automated policy enforcement	72	80
49	Secure coding training via AI simulation	60	55
50	Context-aware security guidance	68	63
51	Vulnerability prediction models	75	70
AI practices for CRSC 6: inadequate encryption			
52	Use of strong encryption algorithms	90	85
53	Secure key management	85	90
54	Implementation of AI-powered code review	75	70
55	Regular security audits and penetration testing	80	65%
56	Automated vulnerability scanning tools	70	60%
57	Developer training in cryptography	65	70%
58	Use of secure libraries and frameworks	85	75%
59	Secure development lifecycle (SDLC) integration	80	70%
60	Threat modeling for encryption risks	75	80%
61	AI-driven monitoring and alerts	70	75%
AI practices for CRSC 7: cross-site scripting (XSS)			
62	Input validation	92	89%
63	Output encoding	85	90%
64	Use of security libraries/frameworks	80	75%
65	Context-aware escaping	85	80%
66	Content security policy (CSP)	75	80%
67	Avoid inline JavaScript	70	65%
68	Sanitize HTML	85	80%
69	Educating developers	80	70%
70	Automated testing tools	70	85%
71	Regular code review	75	70%
72	Secure defaults	55	60%
AI practices for CRSC 8: insufficient logging and monitoring			
73	Automated log analysis	78	85%
74	Contextual logging	80	70%
75	Real-time monitoring with AI	90	85%
76	Dynamic log level adjustment	60	65%
77	Predictive maintenance through log insights	75	80%
78	Correction across multiple systems	88	78%
79	Log enrichment and noise reduction	68	72%
80	Incident root cause analysis	85	83
81	Data privacy and security in logs	65	75
82	Feedback loop for log optimization	62	68
AI Practices for CRSC 9: race conditions			
83	Thread synchronization	92	87
84	Avoid shared state	85	80
85	Race condition testing	80	85
86	Atomic operations	85	78
87	Thread-safe libraries	80	75
88	Deadlock detection and prevention	70	75
89	Code reviews focused on concurrency	75	68
90	Formal verification	65	50
91	Automated tools for race condition detection	75	70
92	AI-assisted static code analysis	78	82
93	Continuous monitoring and feedback loops	70	75
94	Concurrency design pattern	80	78
95	Developer training on concurrency	80	85
AI Practices for CRSC 10: inadequate security testing			
96	Automated vulnerability scanning	75	85
97	AI-driven static code analysis	80	70
98	Dynamic security testing with AI	70	60
99	Predictive threat modeling	80	85
100	Anomaly detection	75	80
101	Continuous AI-base monitoring	60	65
102	AI-powered penetration testing	75	60
103	Adaptive security training	65	75
104	Secure development lifecycle integration	60	75
105	Natural language processing (NLP) for code review	60	70
AI Practices for CRSC 11: supply chain attacks			
106	AI-powered dependency analysis	70	80
107	Automated risk scoring for components	65	75
108	Real-time supply chain monitoring	60	85
109	AI-driven threat intelligence	75	90
110	Behavioral analysis of components	55	70
111	Automated patch management	60	80
112	Provenance verification	65	75
113	Threat simulation and resilience testing	50	70
114	Blockchain and AI Integration	45	60
115	Policy enforcement with AI	76	70
AI Practices for CRSC 12: Insider Threats			
116	AI-based user behavior analytics (UBA)	50	70
117	Role-based access control with AI	70	65
118	AI-driven privilege escalation detection	85	90
119	Automated threat detection models	75	78
120	Continuous monitoring and alerts	70	66
121	AI-augmented employee risk profiling	80	80
122	Sentiment analysis on communication	75	85
123	AI-powered anomaly detection	80	60
124	Dynamic access and authentication systems	85	95
125	Education and awareness programs	70	75
AI Practices for CRSC 13: insecure APIs			
126	Automated API threat detection	76	79
127	Secure code analysis	70	77
128	Behavioral analysis	78	85
129	Encryption validation	74	62
130	API gateway security	62	68
131	Real-time monitoring and alerts	70	60
132	Machine learning for authentication	68	58
133	Input validation and sanitization	65	75
134	AI-powered penetration testing	60	50
135	Policy enforcement via AI	58	72
AI Practices for CRSC 14: Malware in codebase			
136	Static code analysis with AI	83	87
137	Dynamic analysis with AI	78	75
138	Machine learning-based malware detection	90	85
139	Automated code review and bug detection	80	70
140	AI-based threat intelligence	77	82
141	AI-powered code sanitization	65	68
142	Predictive security models	77	74
143	Automated patch generation	73	85
144	Behavioral analysis and anomaly detection	79	72
145	AI-based dependency scanning	83	78
146	AI-driven incident response and remediation	74	87
147	AI for secure development training	68	60
AI Practices for CRSC 15: Compromised CI/CD pipelines			
148	AI-based anomaly detection	85	78
149	Automated threat intelligence integration	80	70
150	Code scanning with AI-powered static and dynamic analysis	90	75
151	AI for secure dependency management	70	80
152	AI for security patch prediction	68	75
153	Behavioral AI for developer access control	84	85
154	AI-driven log analysis for incident response	80	77
155	Automated rollback with AI for compromised builds	78	72
156	AI-powered credential and secrets management	82	88
157	AI-enhanced continuous monitoring and auditing	72	75
158	Adversarial AI simulation for pipeline testing	77	65

### ANN model building

**Fig. 6:**
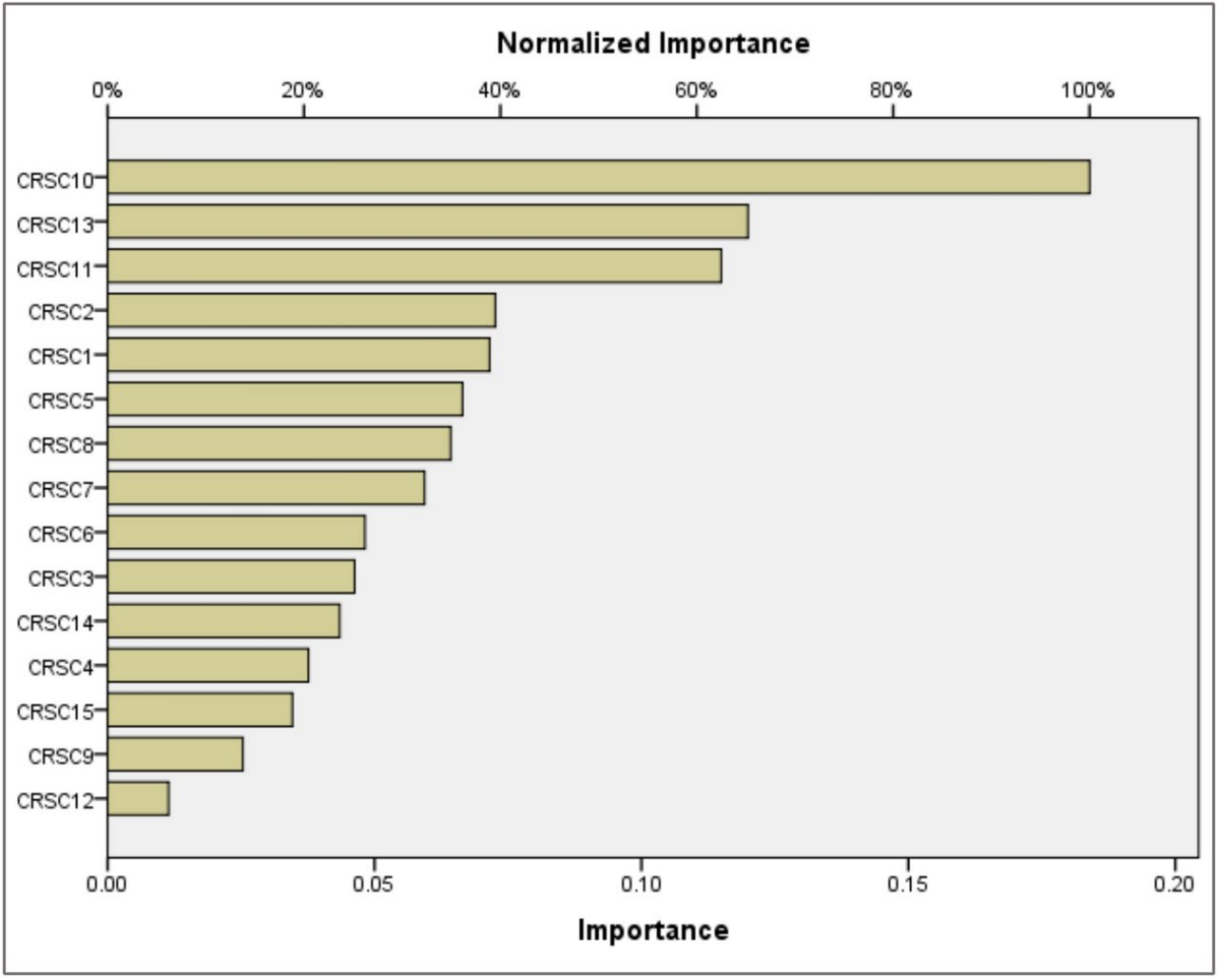
Sensitivity analysis and normalized importance of cybersecurity risks in software coding.

**Fig. 7:**
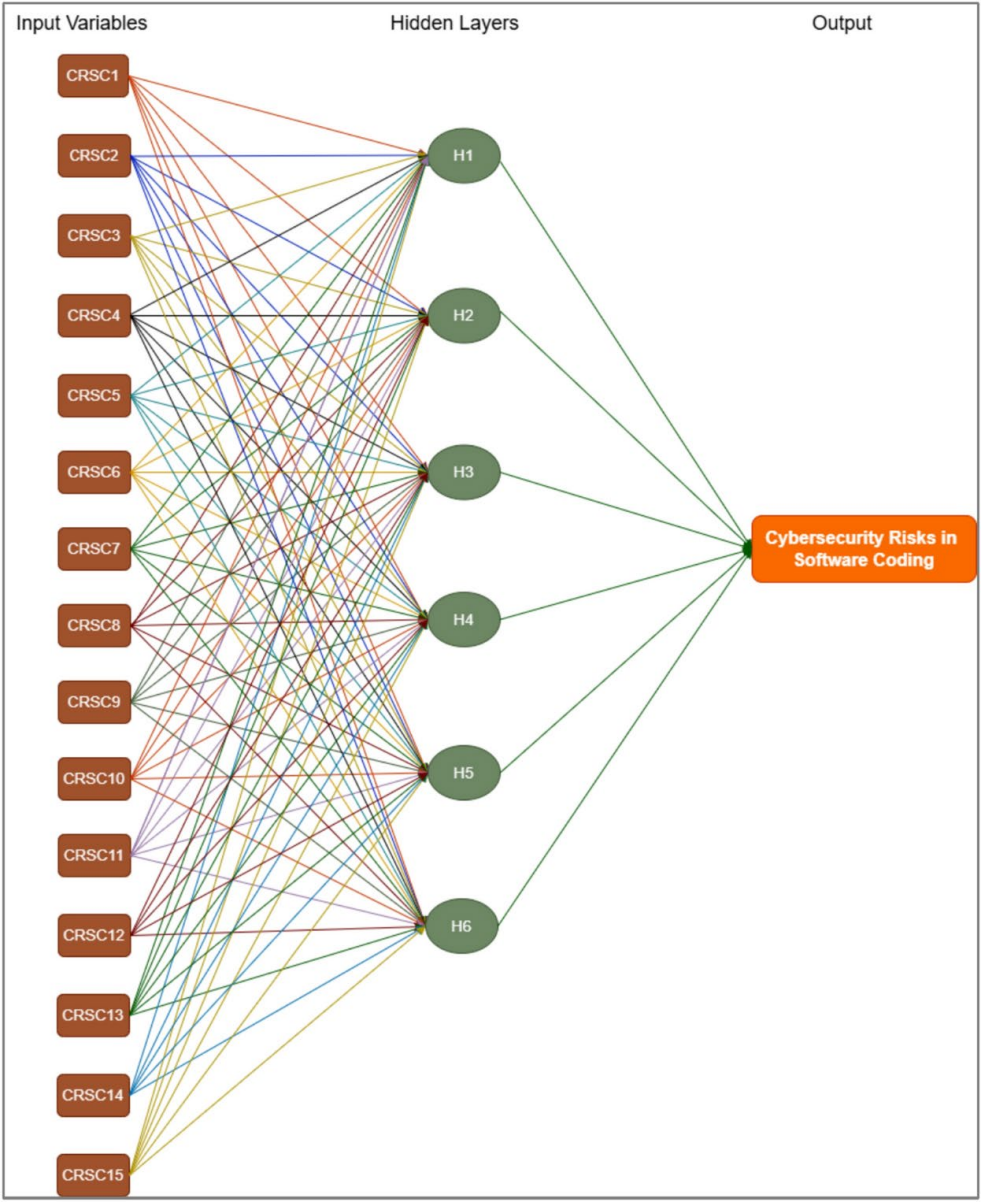
Proposed ANN structure.

**Fig. 8:**
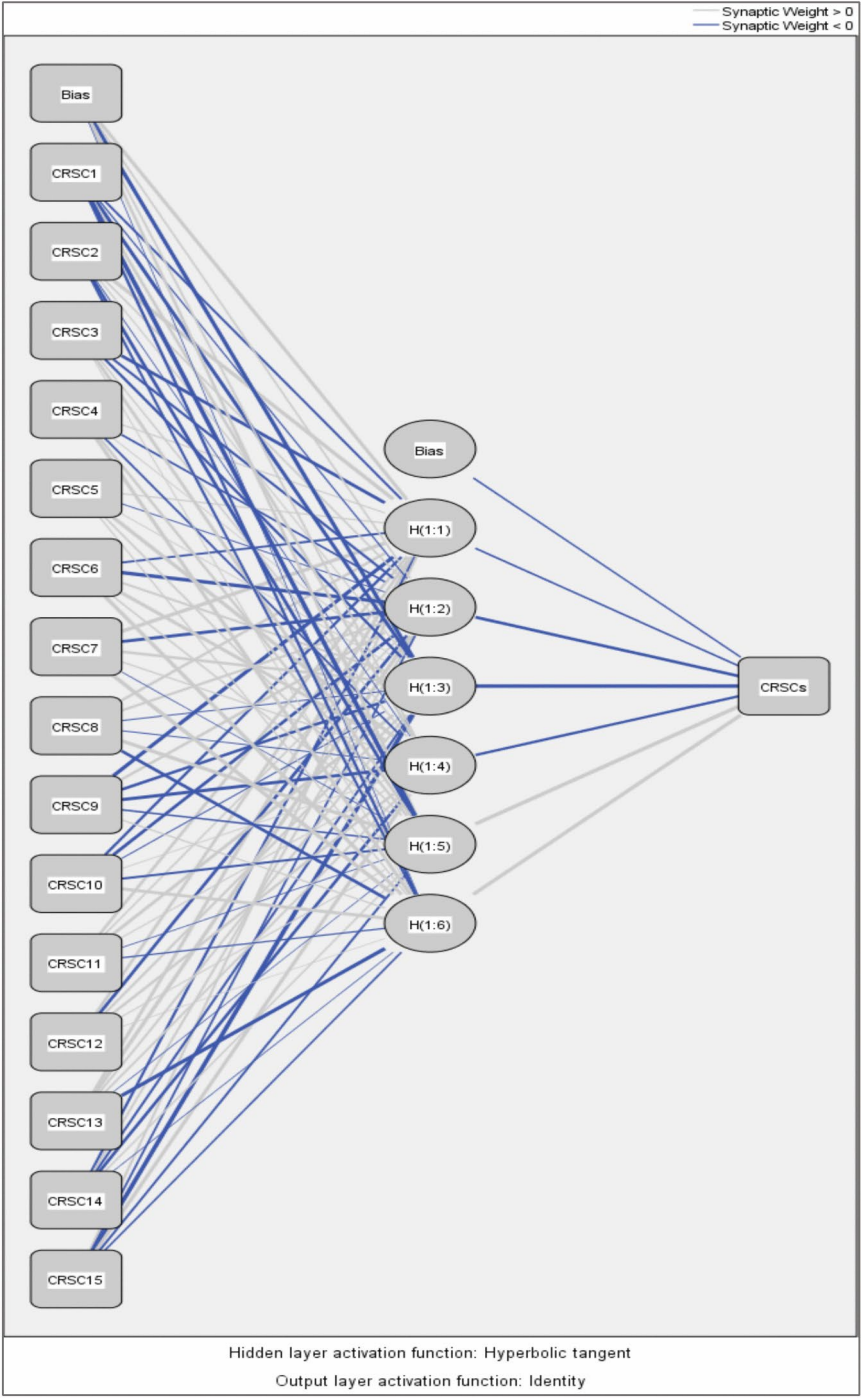
ANN model.

To enhance the clarity and comprehensiveness of the study, we elaborated on the ANN model's training and evaluation process. The dataset comprises 15 distinct vulnerabilities and cybersecurity risks associated with software coding, with 70% allocated for training and 30% for validation to avoid overfitting; tenfold cross-validation was employed to ensure validity. The ANN utilizes the 15 features depicted in Table 4. These features serve as inputs for the input layer of the ANN, while the output layer represents the dependent variable of cybersecurity risks in software coding for analysis. The ANN model was trained using the Adam optimizer with a learning rate of 0.001 for 50 epochs and a batch size 32, utilizing the cross-entropy loss function for classification. The model’s performance was evaluated using Root Mean Square Error (RMSE), yielding a mean RMSE of 0.860 for the testing model and 0.060 for the training model, as presented in Tables 6. Table 7 presents importance and normalized importance of cybersecurity risks in software coding. Figure 8 further illustrates the normalized importance and sensitivity analysis of the cybersecurity risks in software coding, highlighting that the ANN model effectively captures the nonlinear relationships between the independent variables and their effect on these risks. Figures 6 and 7 further highlight the impact of these factors, providing insights into how variations in predicted output values affect the independent variable values. The summary of both the importance and normalized importance of these risks provides an estimate of how changes in predicted output values of the network model impact the independent variable values^71^.

**Table 6:** ANN model summary.

Training	The sum of squares error	0.060
	Relative error	0.009
	Stopping rule used	1 consecutive step(s) with no decrease in error^a^
	Training Time	0:00:00.01
Testing	The sum of Squares error	0.860
	Relative error	3.055

Dependent Variable: Cybersecurity Risks in Software Coding

^a^Error computations are based on the testing sample.

**Table 7:** Importance of cybersecurity risks in software coding.

Independent variable importance			
Code #	Cybersecurity risks in software coding (CRSC)	Importance	Normalized importance (%)
CRSC1	Insecure coding practices	0.072	38.9
CRSC2	Vulnerable dependencies	0.073	39.5
CRSC3	Poor error handling	0.046	25.2
CRSC4	Weak authentication/authorization	0.038	20.5
CRSC5	Misconfigured security controls	0.066	36.1
CRSC6	Inadequate encryption	0.048	26.2
CRSC7	Cross-site scripting (XSS)	0.059	32.3
CRSC8	Insufficient logging and monitoring	0.064	34.9
CRSC9	Race conditions	0.025	13.8
CRSC10	Inadequate security testing	0.184	100.0
CRSC11	Supply chain attacks	0.115	62.5
CRSC12	Insider threats	0.011	6.2
CRSC13	Insecure APIs	0.120	65.2
CRSC14	Malware in codebase	0.043	23.6
CRSC15	Compromised CI/CD pipelines	0.035	18.8

### Results obtained through ISM

ISM is used in research to help structure and clarify complex systems, mainly where multiple variables or factors interact^76,79,99–101^. ISM is beneficial because it allows researchers to break down complex problems into similar elements and to model the relationships between them systematically and hierarchically. ISM can be applied in diverse fields like software development, supply chain management, policy planning, sustainability, and organizational behavior, making it a flexible tool for researchers across disciplines^3,36,82,83^.

We used ISM in this paper to investigate the contextual interrelationship between the security threats in software coding. The contextual relationships among the criteria were established using a Structure-Self-Interaction Matrix (SSIM), which is explained in the following sections:

#### Structural-self-interaction matrix (SSIM)

In the context of cybersecurity vulnerabilities and risks in software coding, the following symbols represent the direction of relationships between a security threat enabler (m) and a node (n):

- ‘V’ indicates a connection between enabler’s m and n.
- ‘A’ indicates a connection between enabler’s n and m.
- ‘X’ indicates that enabler’s m and n interact in the same direction.
- ‘O’ means there is no connection between enabler’s m and n.

Based on expert feedback, we developed the SSIM, which is shown in Table 8.

**Table 8:**
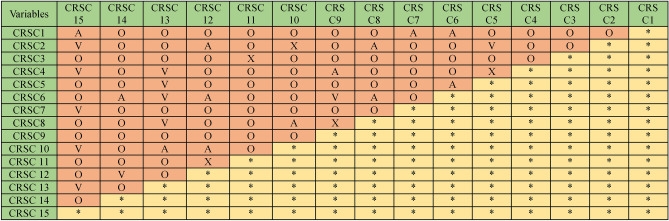
SSIM matrix

With input from industry experts, the ISM methodology was employed to explore the contextual links between key categories of cybersecurity risks in software coding. To gain insights into ISM, we assembled a panel of professionals. Nineteen experts with extensive experience in the field were invited to participate in a preliminary survey and subsequent discussions. These experts came from a diverse array of research institutions and professional backgrounds in software coding. Their feedback was used to construct the SSIM matrix.

Although the sample size was relatively small, limiting the generalizability of the findings, similar studies have been conducted with even fewer experts. For instance, Kannan et al.^100^ used input from five experts to select reverse logistics service providers. Soni et al.^102^ consulted nine experts to study the complexities of urban rail transit systems. Similarly, Attri et al.^103^ used the opinions of five experts to identify key factors for successful, productive maintenance. The interdependencies among DevSecOps challenges categories were analyzed using the ISM method^99^. Other researchers have applied the ISM approach to study DevOps testing^79^ and best test practices^76^.

Table 8 presents cybersecurity risks in software coding associations (CRSC1 to CRSC15) and uses ISM to categorize how these elements are linked. Here’s a step-by-step explanation of what the table signifies:

- Components of the Table:
  - Rows (CRSC 1 to CRSC 15): These may be one or two measures or constituents (which can be cybersecurity risks or aspects about a specific system, like vulnerabilities and cybersecurity risks in software coding or factors in software development organizations).
  - Columns (CRSC1 to CRSC15): These represent the same variables as the rows, showing how one variable relates or interacts with all the other variables.
  - Entries (A, V, X, O): The symbols denote the association or connection among the elements in the rows and columns.
- Interpretation of the Symbols:
  - A (Influence): This means that the variable in the row impacts the variable in the column. For example, in the row marked CRSC1 and column marked CRSC15, the “A” signifies that CRSC1 influences CRSC15.
  - V (Dependency): This suggests that the variable in the column causes the variable in the row. The responses to the dependent variable are presented in the last two columns of the table below. For instance, in row CRSC2 coupled with column CRSC15, a “V” shows that CRSC2 depends on CRSC15.
  - X (Bidirectional Influence): This symbol signifies a feedback loop, suggesting that both variables may be driving the other. For example, the CRSC2 and CRSC10 have an “X,” which only means that these two variables affect each other.
  - (No direct influence): This means that there is no correlation between the two variables given in a row and that of the column. For instance, if we observe row CRSC1 and column CRSC14, there is an “O,” which means that CRSC1 does not control CRSC14 and vice versa.
- Example Interpretation:
  - CRSC15 (Compromised CI/CD Pipelines):
    - Influences: Compromised CI/CD Pipelines CRSC15 with “A”. This is because insecure coding practices can be directed at compromising CI/CD pipelines.
    - Dependent on: The “V” in the columns CRSC2, CRSC4, CRSC7, CRSC10, and CRSC13 show that compromised CI/CD pipelines rely on these factors for service to perform as intended.
    - No Influence: No relation with CRSC3, CRSC5, CRSC6, CRSC8, CRSC9, CRSC11, CRSC12, and CRSC14.
    - Mutual Influences: Another is CRSC2 (Vulnerable Dependencies), labeled “X” with CRSC10 (Inadequate Security Testing), which symbolizes two-way relationships between these two factors.
    - Purpose of the Table:

This table compares the interactions and impacts of cybersecurity risks in software coding based on the typical approach in ISM. By understanding these relationships, researchers can determine:

- Which cybersecurity risks are critically independent (those that control other risks) and frequently dependent (those that are controlled by other risks)?
- Where these risks apply, they help describe how resources and interventions can be appropriately prioritized in software coding.

For example, if CRSC6 (Inadequate Encryption) affects cybersecurity risks such as CRSC8 (Insufficient Logging and Monitoring), CRSC12 (Insider Threats), and CRSC14 (Malware in Codebase) can improve the performance of the entire system. On the other hand, the variable with more significant linkages might need special attention since it tends to be sensitive to other areas.

In summary, this table assists the researcher or practitioner in comprehending several cybersecurity risks in a system and deciding which are vital for the stability of software development organizations.

#### Reachability matrix (Rm)

The reachability matrix was developed by converting the SSIM values (V, A, X, and O) into binary form (0 and 1). The following rules were applied in constructing the matrix:

- When the values between m and n in the SSIM are ‘V’, it is replaced with 1; otherwise, it is set to 0.
- If the value between m and n is ‘A’, it is set to 0, and all other cases are set to 1.
- If the value is ‘X’, it is replaced by 1, and both fields are updated accordingly.
- When m and n have ‘O’ in the SSIM, they are replaced with 0.

The reachability matrix, following these protocols, is represented in Table 9. The transitivity condition is implemented using the value 1*, which corrects any inconsistencies in the SSIM data.

**Table 9:**
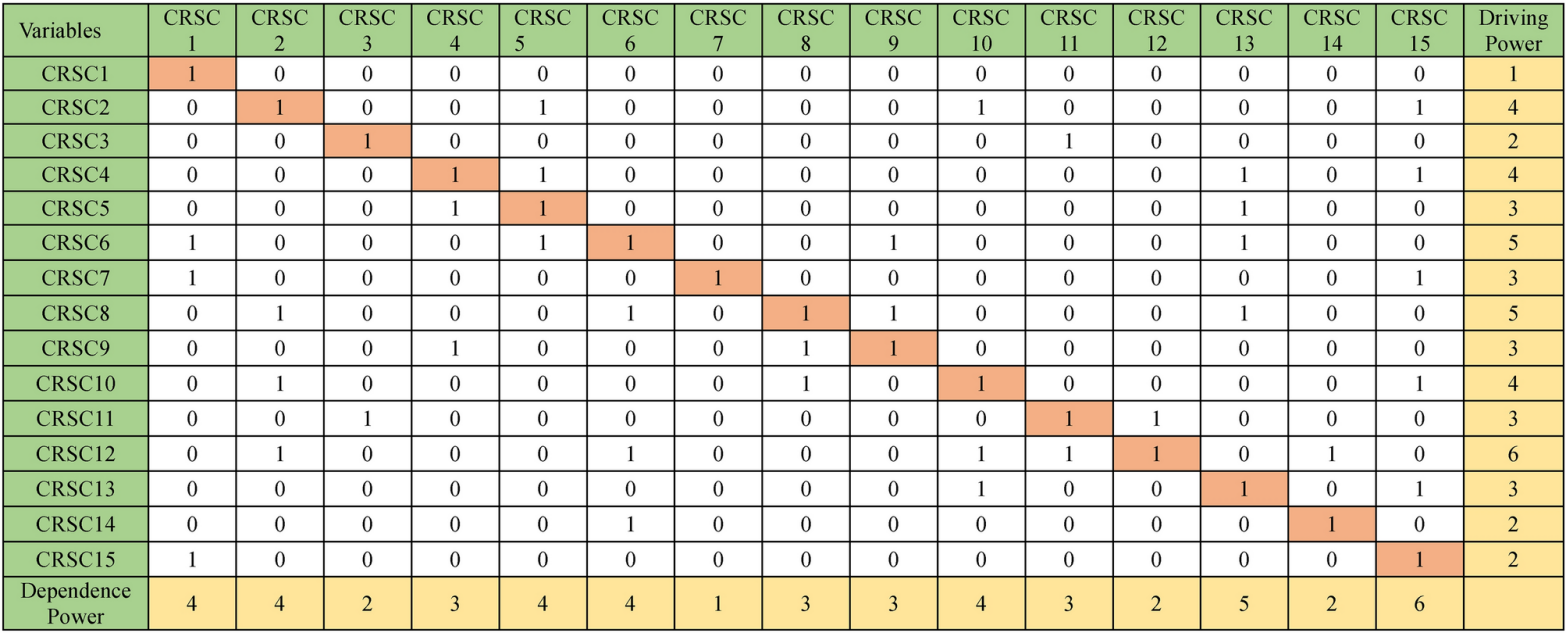
Reachability matrix

Table 10 outlines the new transitivity check and ranks the criteria according to their interdependence and mutual influence. The identified driving force highlights the requirements for each group of cybersecurity risk criteria, while the dependent power reflects the criteria that could contribute to achieving the overall objective. This dependence and influence analysis is valuable for the MICMAC classification, which categorizes the criteria as autonomous, dependent, linking, or independent.

**Table 10:**
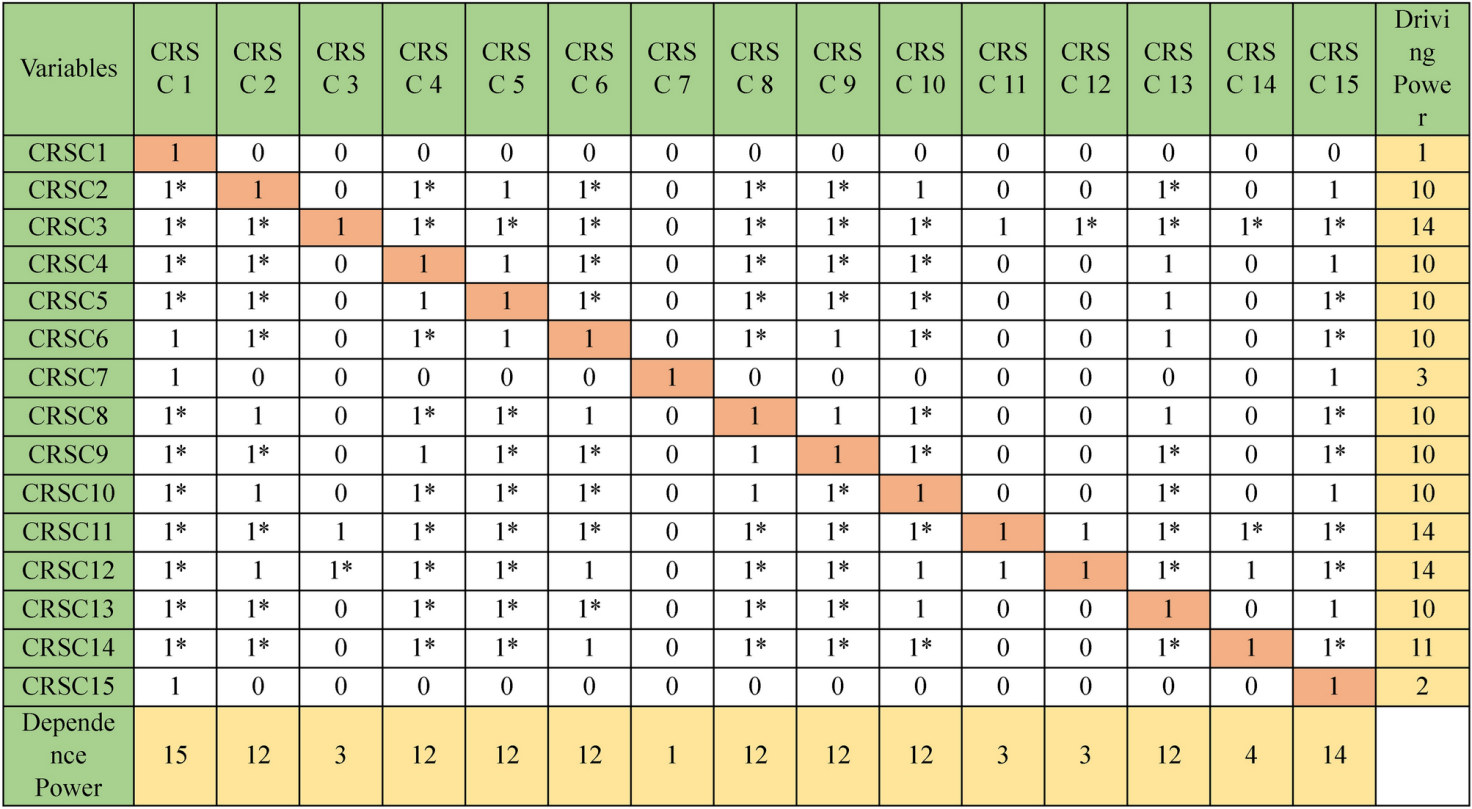
Final reachability matrix

#### Portioning the reachability matrix (Rm)

According to Warfield^104^, a variable’s reachability set includes the variable itself and any other variables contributing to achieving its goal. The intersection of these sets is calculated individually for each component. Elements with the same reachability and intersection sets form the top level of the ISM hierarchy. To progress within the hierarchy, top-level elements must be addressed first. Once identified, the top-level element is removed, and the process is repeated to determine the next level. This iterative approach continues until the hierarchy of all components is established. These levels are critical for constructing the ISM model and diagram. Table 11 presents the 15 criteria categorized as cybersecurity risks in software coding reachability sets, antecedent sets, intersection sets, and corresponding levels.

**Table 11:** Level partitioning of final reachability matrix (FRM).

Level partitions				
Elements (Mi)	Reachability set R(Mi)	Antecedent set A(Ni)	Intersection set R(Mi) ∩ A(Ni)	Levels
Iteration one				
CRSC1	1,	1, 2, 3, 4, 5, 6, 7, 8, 9, 10, 11, 12, 13, 14, 15,	1,	Level-1
CRSC2	1, 2, 4, 5, 6, 8, 9, 10, 13, 15,	2, 3, 4, 5, 6, 8, 9, 10, 11, 12, 13, 14,	2, 4, 5, 6, 8, 9, 10, 13,	…
CRSC3	1, 2, 3, 4, 5, 6, 8, 9, 10, 11, 12, 13, 14, 15,	3, 11, 12,	3, 11, 12,	…
CRSC4	1, 2, 4, 5, 6, 8, 9, 10, 13, 15,	2, 3, 4, 5, 6, 8, 9, 10, 11, 12, 13, 14,	2, 4, 5, 6, 8, 9, 10, 13,	…
CRSC5	1, 2, 4, 5, 6, 8, 9, 10, 13, 15,	2, 3, 4, 5, 6, 8, 9, 10, 11, 12, 13, 14,	2, 4, 5, 6, 8, 9, 10, 13,	…
CRSC6	1, 2, 4, 5, 6, 8, 9, 10, 13, 15,	2, 3, 4, 5, 6, 8, 9, 10, 11, 12, 13, 14,	2, 4, 5, 6, 8, 9, 10, 13,	…
CRSC7	1, 7, 15,	7,	7,	…
CRSC8	1, 2, 4, 5, 6, 8, 9, 10, 13, 15,	2, 3, 4, 5, 6, 8, 9, 10, 11, 12, 13, 14,	2, 4, 5, 6, 8, 9, 10, 13,	…
CRSC9	1, 2, 4, 5, 6, 8, 9, 10, 13, 15,	2, 3, 4, 5, 6, 8, 9, 10, 11, 12, 13, 14,	2, 4, 5, 6, 8, 9, 10, 13,	…
CRSC10	1, 2, 4, 5, 6, 8, 9, 10, 13, 15,	2, 3, 4, 5, 6, 8, 9, 10, 11, 12, 13, 14,	2, 4, 5, 6, 8, 9, 10, 13,	…
CRSC11	1, 2, 3, 4, 5, 6, 8, 9, 10, 11, 12, 13, 14, 15,	3, 11, 12,	3, 11, 12,	…
CRSC12	1, 2, 3, 4, 5, 6, 8, 9, 10, 11, 12, 13, 14, 15,	3, 11, 12,	3, 11, 12,	…
CRSC13	1, 2, 4, 5, 6, 8, 9, 10, 13, 15,	2, 3, 4, 5, 6, 8, 9, 10, 11, 12, 13, 14,	2, 4, 5, 6, 8, 9, 10, 13,	…
CRSC14	1, 2, 4, 5, 6, 8, 9, 10, 13, 14, 15,	3, 11, 12, 14,	14,	…
CRSC15	1, 15,	2, 3, 4, 5, 6, 7, 8, 9, 10, 11, 12, 13, 14, 15,	15,	…
Iteration two				
CRSC1		2, 3, 4, 5, 6, 7, 8, 9, 10, 11, 12, 13, 14, 15,		Level-1
CRSC2	2, 4, 5, 6, 8, 9, 10, 13, 15,	2, 3, 4, 5, 6, 8, 9, 10, 11, 12, 13, 14,	2, 4, 5, 6, 8, 9, 10, 13,	…
CRSC3	2, 3, 4, 5, 6, 8, 9, 10, 11, 12, 13, 14, 15,	3, 11, 12,	3, 11, 12,	…
CRSC4	2, 4, 5, 6, 8, 9, 10, 13, 15,	2, 3, 4, 5, 6, 8, 9, 10, 11, 12, 13, 14,	2, 4, 5, 6, 8, 9, 10, 13,	…
CRSC5	2, 4, 5, 6, 8, 9, 10, 13, 15,	2, 3, 4, 5, 6, 8, 9, 10, 11, 12, 13, 14,	2, 4, 5, 6, 8, 9, 10, 13,	…
CRSC6	2, 4, 5, 6, 8, 9, 10, 13, 15,	2, 3, 4, 5, 6, 8, 9, 10, 11, 12, 13, 14,	2, 4, 5, 6, 8, 9, 10, 13,	…
CRSC7	7, 15,	7,	7,	…
CRSC8	2, 4, 5, 6, 8, 9, 10, 13, 15,	2, 3, 4, 5, 6, 8, 9, 10, 11, 12, 13, 14,	2, 4, 5, 6, 8, 9, 10, 13,	…
CRSC9	2, 4, 5, 6, 8, 9, 10, 13, 15,	2, 3, 4, 5, 6, 8, 9, 10, 11, 12, 13, 14,	2, 4, 5, 6, 8, 9, 10, 13,	…
CRSC10	2, 4, 5, 6, 8, 9, 10, 13, 15,	2, 3, 4, 5, 6, 8, 9, 10, 11, 12, 13, 14,	2, 4, 5, 6, 8, 9, 10, 13,	…
CRSC11	2, 3, 4, 5, 6, 8, 9, 10, 11, 12, 13, 14, 15,	3, 11, 12,	3, 11, 12,	…
CRSC12	2, 3, 4, 5, 6, 8, 9, 10, 11, 12, 13, 14, 15,	3, 11, 12,	3, 11, 12,	…
CRSC13	2, 4, 5, 6, 8, 9, 10, 13, 15,	2, 3, 4, 5, 6, 8, 9, 10, 11, 12, 13, 14,	2, 4, 5, 6, 8, 9, 10, 13,	…
CRSC14	2, 4, 5, 6, 8, 9, 10, 13, 14, 15,	3, 11, 12, 14,	14,	…
CRSC15	15,	2, 3, 4, 5, 6, 7, 8, 9, 10, 11, 12, 13, 14, 15,	15,	Level-2
Iteration three				
CRSC1		2, 3, 4, 5, 6, 7, 8, 9, 10, 11, 12, 13, 14,		Level-1
CRSC2	2, 4, 5, 6, 8, 9, 10, 13,	2, 3, 4, 5, 6, 8, 9, 10, 11, 12, 13, 14,	2, 4, 5, 6, 8, 9, 10, 13,	Level-3
CRSC3	2, 3, 4, 5, 6, 8, 9, 10, 11, 12, 13, 14,	3, 11, 12,	3, 11, 12,	…
CRSC4	2, 4, 5, 6, 8, 9, 10, 13,	2, 3, 4, 5, 6, 8, 9, 10, 11, 12, 13, 14,	2, 4, 5, 6, 8, 9, 10, 13,	Level-3
CRSC5	2, 4, 5, 6, 8, 9, 10, 13,	2, 3, 4, 5, 6, 8, 9, 10, 11, 12, 13, 14,	2, 4, 5, 6, 8, 9, 10, 13,	Level-3
CRSC6	2, 4, 5, 6, 8, 9, 10, 13,	2, 3, 4, 5, 6, 8, 9, 10, 11, 12, 13, 14,	2, 4, 5, 6, 8, 9, 10, 13,	Level-3
CRSC7	7,	7,	7,	Level-3
CRSC8	2, 4, 5, 6, 8, 9, 10, 13,	2, 3, 4, 5, 6, 8, 9, 10, 11, 12, 13, 14,	2, 4, 5, 6, 8, 9, 10, 13,	Level-3
CRSC9	2, 4, 5, 6, 8, 9, 10, 13,	2, 3, 4, 5, 6, 8, 9, 10, 11, 12, 13, 14,	2, 4, 5, 6, 8, 9, 10, 13,	Level-3
CRSC10	2, 4, 5, 6, 8, 9, 10, 13,	2, 3, 4, 5, 6, 8, 9, 10, 11, 12, 13, 14,	2, 4, 5, 6, 8, 9, 10, 13,	Level-3
CRSC11	2, 3, 4, 5, 6, 8, 9, 10, 11, 12, 13, 14,	3, 11, 12,	3, 11, 12,	…
CRSC12	2, 3, 4, 5, 6, 8, 9, 10, 11, 12, 13, 14,	3, 11, 12,	3, 11, 12,	…
CRSC13	2, 4, 5, 6, 8, 9, 10, 13,	2, 3, 4, 5, 6, 8, 9, 10, 11, 12, 13, 14,	2, 4, 5, 6, 8, 9, 10, 13,	Level-3
CRSC14	2, 4, 5, 6, 8, 9, 10, 13, 14,	3, 11, 12, 14,	14,	…
CRSC15		2, 3, 4, 5, 6, 7, 8, 9, 10, 11, 12, 13, 14,		Level-2
Iteration four				
CRSC1		3, 11, 12, 14,		Level-1
CRSC2		3, 11, 12, 14,		Level-3
CRSC3	3, 11, 12, 14,	3, 11, 12,	3, 11, 12,	…
CRSC4		3, 11, 12, 14,		Level-3
CRSC5		3, 11, 12, 14,		Level-3
CRSC6		3, 11, 12, 14,		Level-3
CRSC7				Level-3
CRSC8		3, 11, 12, 14,		Level-3
CRSC9		3, 11, 12, 14,		Level-3
CRSC10		3, 11, 12, 14,		Level-3
CRSC11	3, 11, 12, 14,	3, 11, 12,	3, 11, 12,	…
CRSC12	3, 11, 12, 14,	3, 11, 12,	3, 11, 12,	…
CRSC13		3, 11, 12, 14,		Level-3
CRSC14	14,	3, 11, 12, 14,	14,	Level-4
CRSC15		3, 11, 12, 14,		Level-2
Iteration five				
CRSC1		3, 11, 12,		Level-1
CRSC2		3, 11, 12,		Level-3
CRSC3	3, 11, 12,	3, 11, 12,	3, 11, 12,	Level-5
CRSC4		3, 11, 12,		Level-3
CRSC5		3, 11, 12,		Level-3
CRSC6		3, 11, 12,		Level-3
CRSC7				Level-3
CRSC8		3, 11, 12,		Level-3
CRSC9		3, 11, 12,		Level-3
CRSC10		3, 11, 12,		Level-3
CRSC11	3, 11, 12,	3, 11, 12,	3, 11, 12,	Level-5
CRSC12	3, 11, 12,	3, 11, 12,	3, 11, 12,	Level-5
CRSC13		3, 11, 12,		Level-3
CRSC14		3, 11, 12,		Level-4
CRSC15		3, 11, 12,		Level-2

#### Interpretation of the ISM model

**Fig. 9:**
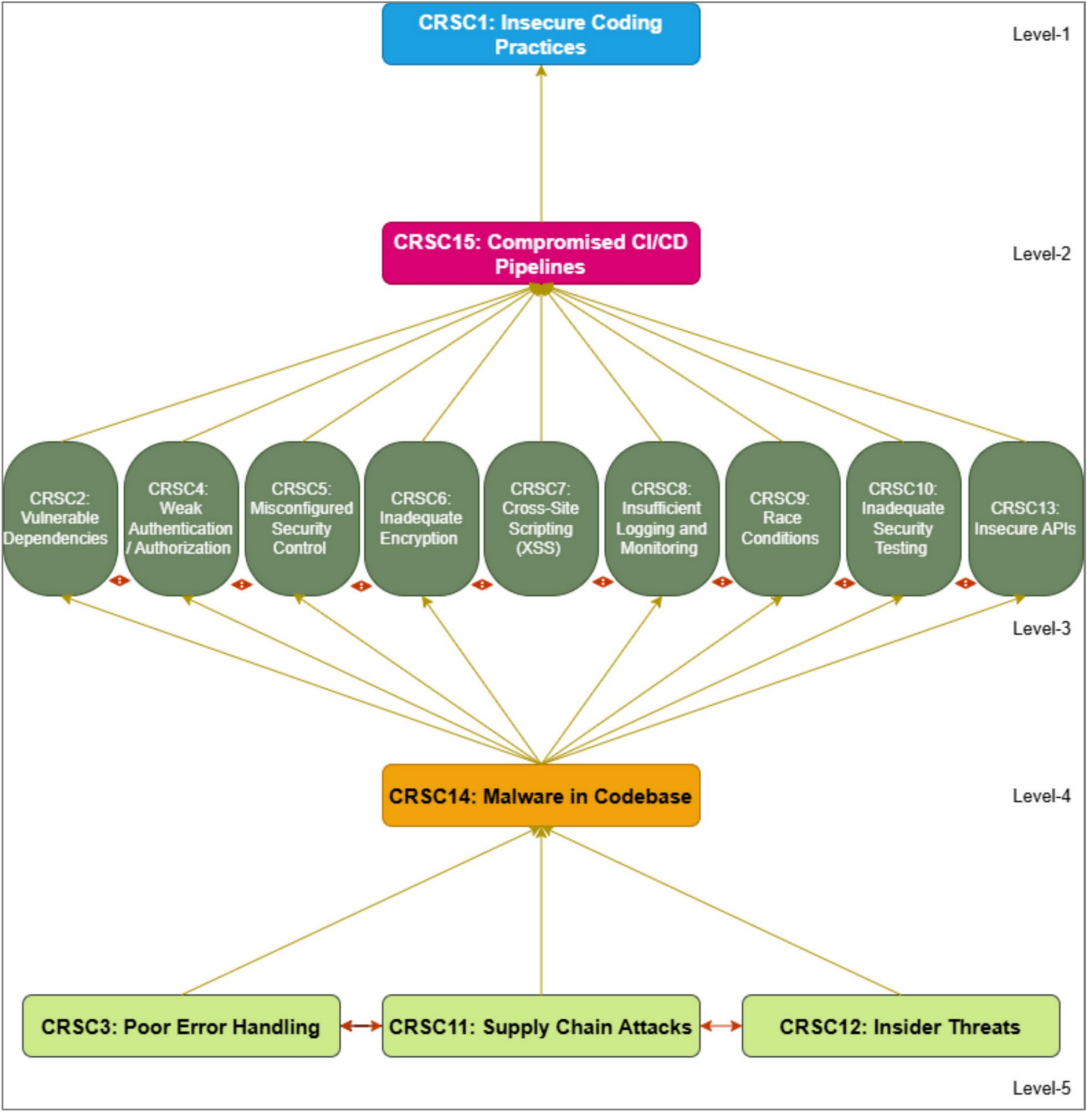
Levels of AI-driven cybersecurity mitigation model for secure software coding.

The ISM model was constructed using the final iteration of the reachability matrix. Arrows between criteria indicate their interdependencies. After converting the digraph into the ISM model (refer to Fig. 9), a transitivity analysis was conducted to resolve any ambiguities in the data.

In Fig. 9:

- Top-Down Approach: The figure outlines how insecure coding practices at the root (Level 1) lead to progressively severe vulnerabilities and threats as we move downward.
- Interconnected Nature: Arrows indicate how vulnerabilities at one level influence or exacerbate issues at subsequent levels.
- Mitigation Objectives: The model demonstrates the importance of systematically addressing each level, from the root (insecure coding practices) to advanced threats (e.g., insider threats and supply chain attacks.)

This figure represents the hierarchical structure of an AI-driven cybersecurity mitigation model aimed at enhancing secure software coding practices. It is organized into five levels, each representing a different aspect of cybersecurity risks and mitigations in the context of software development and deployment:

- Level-1: Insecure Coding Practices (CRSC1)

This represents a fundamental issue at the core of the hierarchy, reflecting insecure coding practices or methodologies that introduce vulnerabilities within the software. It is the primary cause of cybersecurity challenges throughout the SDLC.

- Level-2: Compromised CI/CD Pipelines (CRSC15)

This level emphasizes the vulnerabilities within CI/CD pipelines, which are essential in contemporary software development. Attackers frequently target these pipelines due to the opportunity to introduce malicious code or misconfiguration into production systems. When CI/CD pipelines are compromised, they often reflect underlying insecure coding practices, which can significantly increase risks downstream in the SDLC.

- Level-3: Core Vulnerabilities and Threats

This level identifies specific cybersecurity risks and vulnerabilities that emerge from compromised CI/CD pipelines, such as:

1. CRSC2: The risk increases when you run outdated or insecure third-party sources.
2. CRSC4: The security system fails to control who gets access to sensitive resources.
3. CRSC5: Security tool configurations fail because of incorrect setup designs.
4. CRSC6: Sensitive data needs strong encryption protection, but the organization lacks it.
5. CRSC7: Attackers exploit vulnerabilities inside client-side systems.
6. CRSC8: Few user activity records and system monitoring tools can identify trouble signals.
7. CRSC9: When timing vulnerabilities let hackers break into running programs, race conditions develop.
8. CRSC13: Systems with weak endpoints fail to protect online connections with external programs.

- Level-4: Malware in Codebase (CRSC14)

Level 3 vulnerabilities allow attackers to add harmful code to the product at Level 4. When malicious code enters the codebase, it becomes a starting point for more significant security concerns by threatening software trustworthiness and user confidence.

- Level-5: Advanced Threats

This level encapsulates high-level threats that stem from malware or vulnerabilities in the software codebase:

1. CRSC3: Unpredicted errors let sensitive system information escape.
2. CRSC11: Supply Chain Attacks happen when criminals break into third-party suppliers or upstream providers of system parts.
3. CRSC12: Malicious or negligent actions by trusted individuals within the organization.

Figure 8 illustrates a comprehensive and structured approach to identifying and mitigating crosscutting security threats in software development and AI-driven methodology. Organizations can significantly improve their cybersecurity posture and software development practices by addressing vulnerabilities at each level.

#### MICMAC analysis

**Fig. 10:**
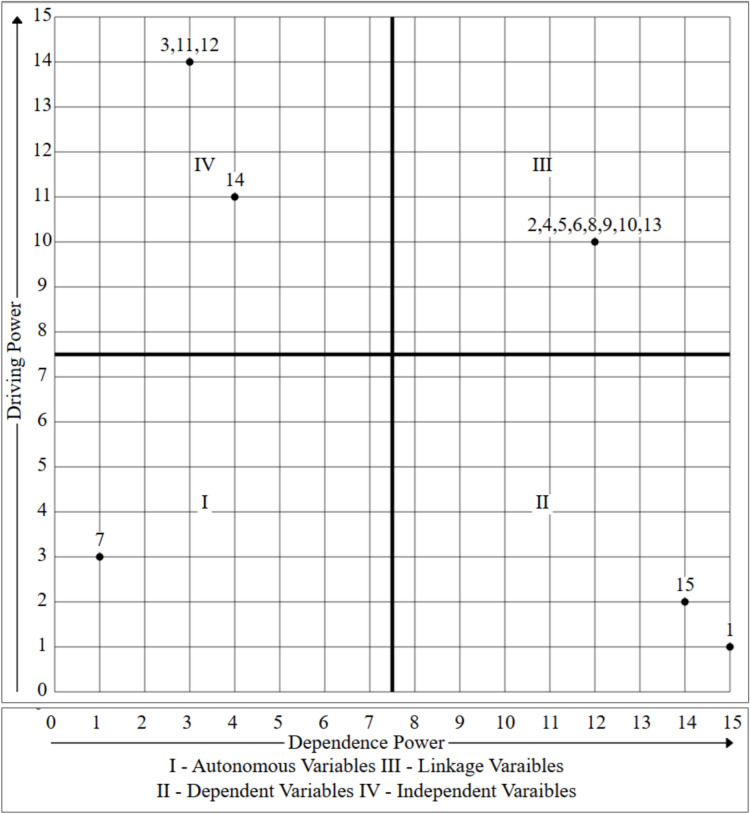
Graphical view of MICMAC analysis.

MICMAC stands for matrix cross-impact matrix classification. The MICMAC analysis examines the key aspects (categories) of the system. According to Attri et al.^103^, MICMAC involves creating a graph that categorizes factors based on their driving and dependence power. MICMAC analysis is used to classify these factors and validate the interpretive structural model’s findings to support conclusions in the study^70^. Enablers are grouped into four categories (see Fig. 10). This classification helps identify the role of various variables in a system, whether independent, dependent, autonomous, or linkage variables.

Figure 9 demonstrates a MICMAC of cybersecurity risks to software coding, in which the Driving Power and the Dependence Power have been used to classify the risks. This method assists in categorizing variables to evaluate the effects of a variable and its dependency on other components in a system.

Figure 10 plots variables on two axes:

- Driving Power (Vertical Axis): Indicates how strongly a variable influences other variables. High driving power means the variable has a more significant impact on the system.
- Dependence Power (Horizontal Axis): Indicates how much other variables influence a variable. High dependence power means the variable is more reliant on others.

The variables (cybersecurity risks) are distributed into four quadrants based on their driving and dependence powers:

- Quadrants and Variable Classification:
  - *Quadrant I: Autonomous Variables*
    - Low Driving Power and Low Dependence Power
    - Represented here by CRSC7 (Cross-Site Scripting).
    - These variables are weakly connected to the system and have minimal influence or dependence.
    - They are often considered relatively insignificant in the system.
  - *Quadrant II: Dependent Variables*
    - Low Driving Power and High Dependence Power
    - Represented here by CRSC1 and CRSC15.
    - Others heavily influence these variables but do not significantly influence other variables.
    - They are often outcomes of changes in other parts of the system.
  - *Quadrant III: Linkage Variables*
    - High Driving Power and High Dependence Power
    - Represented here by CRSC2, CRSC4, CRSC5, CRSC6, CRSC8, CRSC9, CRSC10, and CRSC13.
    - These variables are highly interdependent and serve as a link in the system.
    - Changes to these variables can lead to cascading effects as causes and outcomes.
    - They are unstable and need careful management.
  - *Quadrant IV: Independent Variables*
    - High Driving Power and Low Dependence Power
    - Represented here by CRSC3, CRSC11, CRSC12 and CRSC14.
    - These variables strongly influence the system but are minimally affected by other variables.
    - They are key system drivers and often represent the root causes or critical leverage points.
- Insights from the Fig. 10:
  1. Key Drivers (Independent Variables):
    Cybersecurity risks CRSC3, CRSC11, CRSC12, and CRSC14 are crucial to focus on, as they have high driving power and shape the entire system's behavior.
  2. Highly Interdependent Variables (Linkage):
    Cybersecurity risks in Quadrant III (CRSC2, CRSC4, CRSC5, CRSC6, CRSC8, CRSC9, CRSC10, CRSC13) need careful monitoring, as they can create feedback loops and instability in the system due to their high interdependence.
  3. Dependent Variables (Quadrant II):
    Cybersecurity CRSC1 and CRSC15 are outcomes influenced by changes elsewhere in the system. Managing the drivers will indirectly control these variables.
  4. Autonomous Variables (Quadrant I):
    Cybersecurity CRSC7 has minimal influence and dependence. It may not play a critical role in the current system dynamics but should still be monitored for context.

The MICMAC analysis helps identify critical drivers of success or risk in organizational strategies. It understands which factors drive systemic change and which are merely outcomes. It also prioritizes areas to intervene for maximum impact on the overall system.

#### Development of conical matrix (CM)

The primary purpose of MICMAC analysis is to develop a conical matrix. The data from Tables 10 and 11 were utilized to construct the conical matrix shown in Table 12. The driving and dependence power for each criterion, labeled as “Dri” and “Dep,” is outlined in Table 9. First, their levels organized the requirements (as shown in Table 12). Then, the values of each criterion were compared based on the information in Table 9.

**Table 12:**
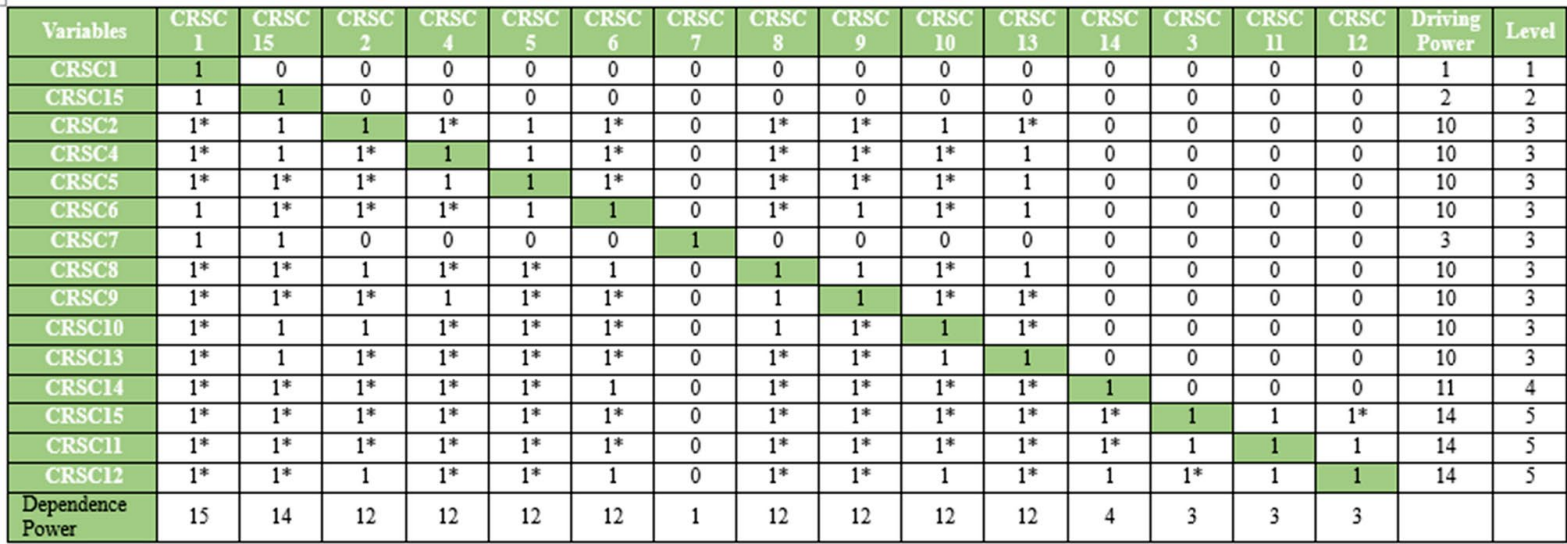
Conical matrix (CM).

## Development of AI-driven cybersecurity mitigation model for secure software coding: using ANN-ISM approach

The proposed AI-driven cybersecurity mitigation model for secure software coding is derived from the frameworks of SAMM^105^, BSIMM^106^, and SCCCMM^107^. Five levels were adapted to create the proposed model using these models as a foundation, each consisting of various process areas. Figure 10 depicts the general procedure for making the proposed model. The following steps were performed for the development of the AI-driven cybersecurity mitigation model for secure software coding:

- Data collection
  - ANN Data: We combine data from a survey of academic sources plus real-world studies to create a strong database to train the ANN system. We normalize and prepare our data to make it consistent and exact in its measurement.
  - ISM Data: To understand how cybersecurity risks affect software coding, we conducted surveys and focus groups through online interviews with experts.
- Model building
  - Training the ANN: The trained ANN depends on processing qualitative information for the model. The neural network system uses input data relationships to predict cybersecurity risk conditions that benefit each software coding system.
  - Constructing ISM: Qualitative data becomes the foundation for designing an ISM chart that depicts all security risk influences on software coding security.
- Model integration
  - Hybrid Framework: Our team creates one unified approach that combines ANN results with ISM findings. ANN helps us forecast threats and find secure settings, but ISM clearly shows us how different cybersecurity risks work together with their primary components.
  - Model Validation: Our integrated model goes through multiple tests with diverse datasets and security experts to show that it helps spot and solve coding security problems in software.
  - Implementation: The validated model helps prevent software coding problems by joining ANN predictions with ISM analysis to create complete security protection methods for coding projects.

**Fig. 11:**
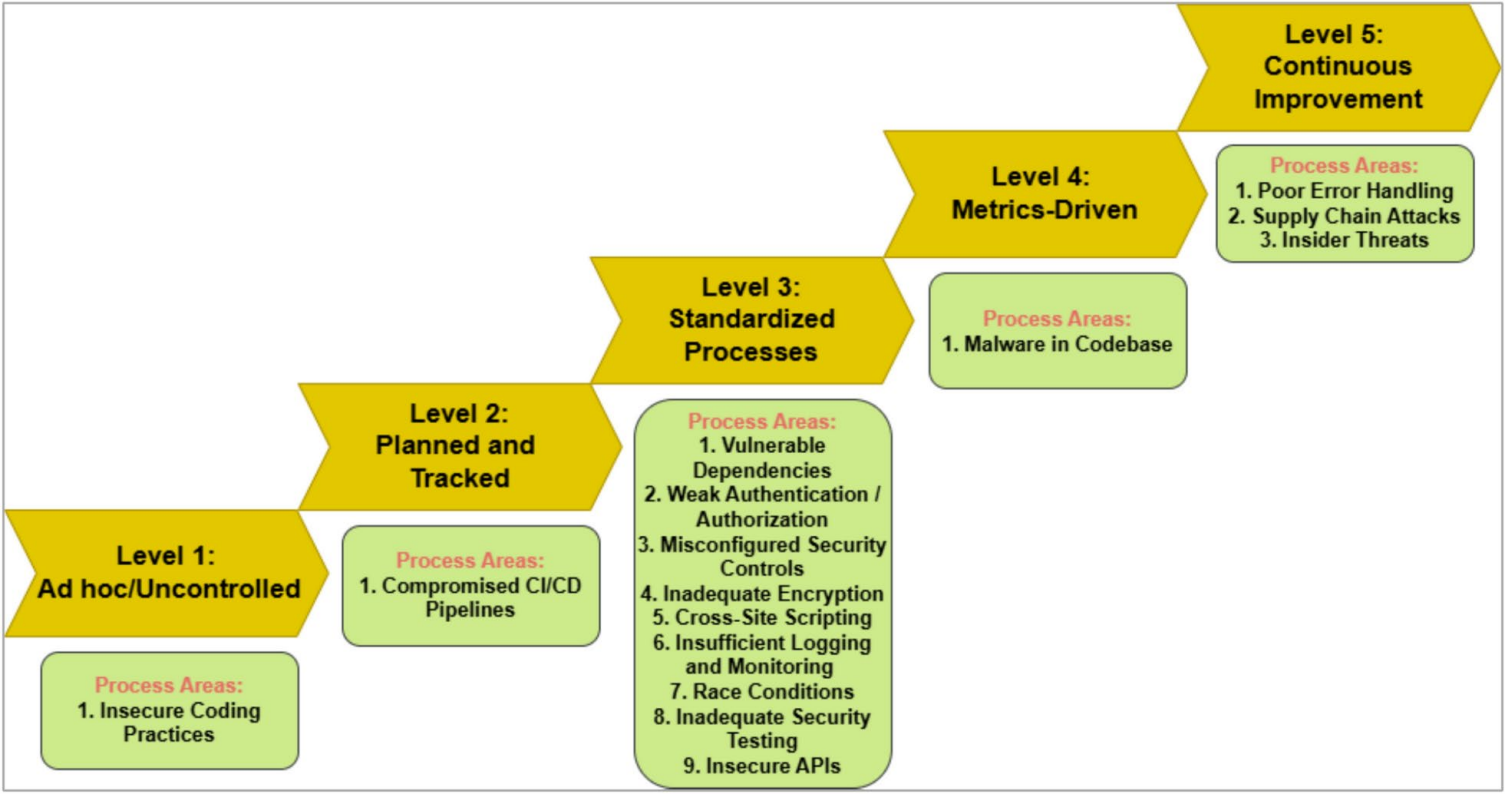
AI-driven cybersecurity mitigation model for secure software coding.

Figure 11 shows our AI-driven model to protect software coding systems using ANN-ISM, which offers five tiers of growing sophistication to tackle cybersecurity risks. Every level has its own process areas responsible for protecting the software coding. The following subsections present the breakdown analysis and significance of each level:

Figure 11 depicts an AI-driven cybersecurity Mitigation Model for Secure Software Coding, with different levels representing increasing maturity and security sophistication. The following subsections describe more explanation of the figure:

### Level-1: Ad hoc/uncontrolled

Processes lack structure or consistency, and security is typically reactive. Coding practices are unregulated, leaving systems vulnerable to threats.

- Process areas
  - Insecure coding practices: Developers follow no secure coding standards, leading to easily exploitable vulnerabilities.

### Level-2: planned and tracked

Organizations at this level begin implementing planned processes with tracking mechanisms, but these are still basic and often insufficient for advanced threats. Experts take specific steps to counter threats, yet their efforts remain scattered across the cybersecurity landscape.

- Process areas
  - Compromised CI/CD pipelines: Reviewers see CI/CD pipelines as ways attackers can break into systems unless organizations implement proper security first. Attackers use pipeline weak points to inject harmful software into the system.

### Level-3: standardized processes

Despite unified security procedures, using defective third-party elements remains resistant to organizations. Inadequate identity controls and deficient testing cause additional problems.

- Process areas
  - Vulnerable Dependencies
  - Weak Authentication/Authorization
  - Misconfigured Security Controls
  - Inadequate Encryption
  - Cross-Site Scripting (XSS)
  - Insufficient Logging and Monitoring
  - Race Conditions
  - Inadequate Security Testing
  - Insecure APIs

This stage includes a broader spectrum of vulnerabilities that require systematic identification and remediation. Failure to address these issues can lead to data breaches or service disruptions.

### Level-4: metrics driven

Measurable data supports the process of choosing proper security measures. Despite these advances, malware injection problems continue due to external library use or insider threats.

- Process Areas
  - Malware in Codebase: An undetected malware infection can lead to unauthorized data leaks plus system failures, with hackers receiving unauthorized control.

### Level-5: continuous improvement

Software development organizations dedicate ongoing energy to making their security standards better. The remaining security threats include weak protection against errors plus problems from supply chain partners and trusted employees.

- Process areas
  - Poor Error Handling
  - Supply Chain Attacks
  - Insider Threats

Because of its intricacy, the software security system needs sophisticated detection and response methods. The system should show what happens inside it while reacting fast to security problems to make the platform more trustworthy.

The AI-based cybersecurity Mitigation Model shows how going from essential reactive protection at Level 1 to refined ongoing improvement at Level 5 creates a secure coding system. The model requires companies to link security measures with their current development process level. New teams primarily focus on critical security concepts such as secure programming and the security of their continuous integration/delivery platforms. The intermediate security setup steps cover multiple significant concerns, including secure encryption of dependencies and secured API access. The later security stages depend on metrics to enhance defenses against malicious programs and internal data breaches through supply chains.

Organizations developing secure software must invest their resources in building their security maturity step by step. Early problem selection and resolution protect against future system risk accumulation, leading to enhanced performance standards over time.

### Model evaluation

The proposed AI-driven cybersecurity Mitigation Model for Secure Software Coding is structured into four evaluation levels, as follows:

1. Beginner: At this level, the organization focuses on testing for vulnerabilities in its software. The qualitative score for this level ranges from 0 to 15%.
2. Understanding: This level involves documenting and implementing cybersecurity risks in software coding. The qualitative score falls from 15 to 50%.
3. Improvement: This level focuses on automating processes and improving software development organizations in the coding process. The qualitative score ranges from 50 to 85%.
4. Advanced: At this stage, the organization is reviewing, optimizing, and further developing its cybersecurity process in software coding. The qualitative score ranges from 85 to 100%.

To assess the effectiveness of our process domain and practices, we adapted the SCAMPI^42^ method. The proposed model is based on the evaluation scale by IBM’s Rational Unified Process (RUP) division, as shown in Table 13. The IBM RUP scale uses numerical values, where 0 indicates no experience or knowledge, and 3 represents complete mastery. Each mitigation practice is scored, and the median value (50) is used to calculate the average for that category. The median score for each category is then used to determine the level of development within that category. Our model’s median always corresponds to a whole number that fits into one of IBM’s four RUP levels, ensuring no overlap between mitigation levels. This approach maintains a clear distinction between the different stages of maturity, preserving the integrity of the model’s maturity assessment.

**Table 13:** Mitigation levels according to RUP defined by IBM.

S. No	Range value in % by IBM	Range of median value for AI-driven cybersecurity mitigation model for secure software coding	Mitigation level
1	0–15%	0 < Median ≤ 0.45	Beginner
2	15–50%	0.45 < Median ≤ 1.5	Understanding
3	50–85%	1.5 < Median ≤ 2.55	Improvement
4	85–100%	2.55 < Median ≤ 3	Advanced

The Software Engineering Research Group (SERG) University, Malakand, Pakistan, pilot-tested the proposed model. Four professors participated, including one full, two associate professors, and one assistant. An input to the task was a document about the proposed model structure, for which the experts were asked to give suggestions. When completing a set of questions to evaluate the model's structure, each expert provided the following responses, the analysis of which is presented in Table 14.

**Table 14:** Evaluation of AI-driven cybersecurity mitigation model for secure software coding in academia (SERG_UOM).

S. No	Structure	Agree		Disagree		Undecided	
		N	%	N	%	N	%
1	Each level of AI-driven cybersecurity mitigation model for secure software coding is clear and requires no explanation	4	100	0	0	0	0
2	Each AI-driven cybersecurity mitigation model for secure software coding level is feasible for software coding security	3	75	0	0	1	25
3	The AI-driven cybersecurity mitigation model for secure software coding framework can be used to pinpoint where an organization has room to improve its cybersecurity risks	4	100	0	0	0	0
4	It is helpful to divide cybersecurity risks in software coding into different levels	3	75	1	25	0	0
5	The 5GN-SM AI-driven cybersecurity mitigation model for secure software coding five levels are useful	2	50	1	25	1	25

This paper presents a case study with a well-established security network organization as a practical assessment of the proposed model. Other study participants include the company’s software security quality assurance and network configuration team leaders. The case study was conducted based on the information that we presented to the members of our research team, along with the documents that were needed. Researchers in software engineering widely apply such case study methodology to collect and analyze data^57,108–112^. To coordinate all the information, an Excel checklist of the proposed model categories, processes, and practices was made. The results of the evaluation, conducted by the company’s team, are summarized (see Table 15) below based on the identified mitigation levels:

- This company uses conventional techniques, and security network configuration is not their priority.
- The company has a documented security network configuration process.
- There is a scope to enhance network security by automating some processes or providing more efficient ways.
- The company has extensively tried to enhance and elaborate on secure network configuration techniques.

**Table 15:** Example of a case study for evaluation of AI-driven cybersecurity mitigation model for secure software coding.

ID	Process areas (PA)	Mitigation levels			
Level 1	Ad hoc/Uncontrolled	Beginner (0)	Understanding (1)	Improvement (2)	Advanced (3)
PA 1	Insecure coding practices				
Practice 1	Automated code analysis tools			2	
Practice 2	Secure coding standards enforcements		1		
Practice 3	Threat modeling and risk assessment				3
Practice 4	AI-powered code reviews		1		
Practice 5	Secure framework recommendations				3
Practice 6	Training and education			2	
Practice 7	Monitoring and logging automation	0			
Practice 8	CI/CD pipeline security integration				3
Practice 9	Real-time vulnerability patching			2	
Practice 10	Phishing and social engineering AI				3
Outcome of appraisal for AI-driven cybersecurity mitigation model for secure software coding level-1 (PA1: insecure coding practices) practices covered by the company		Score		3	
		Mitigation level		Advanced	

Level 2	Planned and tracked				
PA 1	Compromised CI/CD Pipelines				
Practice 1	AI-Based anomaly detection		1		
Practice 2	Automated threat intelligence integration			2	
Practice 3	Code Scanning with AI-powered static and dynamic analysis				3
Practice 4	AI for Secure dependency management		1		
Practice 5	AI for security patch prediction				3
Practice 6	Behavioral AI for developer access control			2	
Practice 7	AI-driven log analysis for incident response				3
Practice 8	Automated rollback with AI for compromised builds		1		
Practice 9	AI-powered credential and secrets management			2	
Practice 10	AI-enhanced continuous monitoring and auditing				3
Practice 10	Adversarial AI simulation for pipeline testing	0			
Outcome of appraisal for AI-driven cybersecurity mitigation model for secure software coding Level-2 (PA1: compromised CI/CD Pipelines) practices covered by the company		Score		3	
		Mitigation level		Advanced	

Level 3	Standardized processes				
PA 1	Vulnerable dependencies				
Practice 1	Automated dependency scanning		1		
Practice 2	Version control and dependency updates			2	
Practice 3	Risk prioritization using AI models				3
Practice 4	Natural language processing for vulnerability analysis	0			
Practice 5	Machine learning for vulnerability prediction		1		
Practice 6	Dependency mapping and visualization				3
Practice 7	Secure coding guidelines recommendation			2	
Practice 8	Continuous integration and continuous deployment (CI/CD)	0			
Practice 9	Dynamic vulnerability testing			2	
Practice 10	AI-enhanced software composition analysis (SCA)			2	
Outcome of appraisal for AI-driven cybersecurity mitigation model for secure software coding level-3 (PA1: vulnerable dependencies) practices covered by the company		Score		2	
		Mitigation Level		Improvement	

Level 3	Standardized processes				
PA 2	Weak Authentication/Authorization				
Practice 1	Enforce strong password policies				3
Practice 2	Implement multi-factor authentication				3
Practice 3	Role-based access control (RBAC)			2	
Practice 4	Use secure token-based authentication		1		
Practice 5	Input validation and sanitization			2	
Practice 6	Implement session management controls				3
Practice 7	Adopt secure Communication protocols				3
Practice 8	Regular security testing	0			
Practice 9	Centralized identify management		1		
Practice 10	Monitor and log authentication events			2	
Outcome of appraisal for AI-driven cybersecurity mitigation model for secure software coding level-3 (PA2: weak authentication/ authorization) practices covered by the company		Score		3	
		Mitigation level		Advanced	

Level 3	Standardized processes				
PA3	Misconfigured security controls				
Practice 1	Automated security scanning				3
Practice 2	Real-time code analysis			2	
Practice 3	Intelligent configuration templates		1		
Practice 4	Anomaly detection in code behavior	0			
Practice 5	Continuous learning and updates				3
Practice 6	Role-based access recommendations			2	
Practice 7	Automated policy enforcement		1		
Practice 8	Secure coding training via AI simulation				3
Practice 9	Context-aware security guidance		1		
Practice 10	Vulnerability prediction models				3
Outcome of appraisal for AI-driven cybersecurity mitigation model for secure software coding level-3 (PA3: misconfigured security controls) practices covered by the company		Score		3	
		Mitigation level		Advanced	

Level 3	Standardized processes				
PA4	Inadequate encryption				
Practice 1	Use of strong encryption algorithms				3
Practice 2	Secure key management			2	
Practice 3	Implementation of AI-powered code review				3
Practice 4	Regular security audits and penetration testing		1		
Practice 5	Automated vulnerability scanning tools			2	
Practice 6	Developer training in cryptography	0			
Practice 7	Use of secure libraries and frameworks			2	
Practice 8	Secure development lifecycle (SDLC) integration				3
Practice 9	Threat modeling for encryption risks			2	
Practice 10	AI-driven monitoring and alerts		1		
Outcome of appraisal for AI-driven cybersecurity mitigation model for secure software coding level-3 (PA4: inadequate encryption) practices covered by the company		Score		2	
		Mitigation level		Improvement	

Level 3	Standardized processes				
PA5	Cross-site scripting (XSS)				
Practice 1	Input validation			2	
Practice 2	Output encoding				3
Practice 3	Use of security libraries/frameworks		1		
Practice 4	Context-aware escaping	0			
Practice 5	Content security policy (CSP)			2	
Practice 6	Avoid inline JavaScript		1		
Practice 7	Sanitize HTML				3
Practice 8	Educating developers		1		
Practice 9	Automated testing tools				3
Practice 10	Regular code review			2	
Practice 11	Secure defaults				3
Outcome of appraisal for AI-driven cybersecurity mitigation model for secure software coding level-3 (PA5: XSS) practices covered by the company		Score		3	
		Mitigation level		Advanced	

Level 3	Standardized processes				
PA6	Insufficient logging and monitoring				
Practice 1	Automated log analysis				3
Practice 2	Contextual logging			2	
Practice 3	Real-time monitoring with AI				3
Practice 4	Dynamic log level adjustment		1		
Practice 5	Predictive maintenance through log insights			2	
Practice 6	Correction across multiple systems			2	
Practice 7	Log enrichment and noise reduction				3
Practice 8	Incident root cause analysis			2	
Practice 9	Data privacy and security in logs		1		
Practice 10	Feedback loop for log optimization	0			
Outcome of appraisal for AI-driven cybersecurity mitigation model for secure software coding level-3 (PA6: insufficient logging and monitoring) practices covered by the company		Score		2	
		Mitigation level		Improvement	

Level 3	Standardized processes				
PA7	Race conditions				
Practice 1	Thread synchronization				3
Practice 2	Avoid shared state			2	
Practice 3	Race condition testing				3
Practice 4	Atomic operations			2	
Practice 5	Thread-safe libraries				3
Practice 6	Deadlock detection and prevention		1		
Practice 7	Code reviews focused on concurrency	0			
Practice 8	Formal verification			2	
Practice 9	Automated tools for race condition detection			2	
Practice 10	AI-assisted static code analysis		1		
Practice 11	Continuous monitoring and feedback loops				3
Practice 12	Concurrency design pattern				3
Practice 13	Developer training on concurrency		1		
Outcome of appraisal for AI-driven cybersecurity mitigation model for secure software coding level-3 (PA7: race conditions) practices covered by the company		Score		3	
		Mitigation level		Advanced	

Level 3	Standardized processes				
PA8	Inadequate security testing				
Practice 1	Automated vulnerability scanning				3
Practice 2	AI-driven static code analysis			2	
Practice 3	Dynamic security testing with AI		1		
Practice 4	Predictive threat modeling	0			
Practice 5	Anomaly detection				3
Practice 6	Continuous AI-base monitoring		1		
Practice 7	AI-powered penetration testing				3
Practice 8	Adaptive security training			2	
Practice 9	Secure development lifecycle integration			2	
Practice 10	Natural language processing (NLP) for code review			2	
Outcome of appraisal for AI-driven cybersecurity mitigation model for secure software coding level-3 (PA8: inadequate security testing) practices covered by the company		Score		2	
		Mitigation level		Improvement	

Level 3	Standardized processes				
PA9	Insecure APIs				
Practice 1	Automated API threat detection			2	
Practice 2	Secure code analysis				3
Practice 3	Behavioral analysis			2	
Practice 4	Encryption validation				3
Practice 5	API gateway security			2	
Practice 6	Real-time monitoring and alerts	0			
Practice 7	Machine learning for authentication			2	
Practice 8	Input validation and sanitization		1		
Practice 9	AI-powered penetration testing		1		
Practice 10	Policy enforcement via AI				3
Outcome of appraisal for AI-driven cybersecurity mitigation model for secure software coding level-3 (PA9: insecure APIs) Practices covered by the company		Score		2	
		Mitigation level		Improvement	

Level 4	Metrics driven				
PA1	Malware in codebase				
Practice 1	Static code analysis with AI			2	
Practice 2	Dynamic analysis with AI		1		
Practice 3	Machine learning-based malware detection				3
Practice 4	Automated code review and bug detection			2	
Practice 5	AI-based threat intelligence				3
Practice 6	AI-powered code sanitization	0			
Practice 7	Predictive security models		1		
Practice 8	Automated patch generation				3
Practice 9	Behavioral analysis and anomaly detection		1		
Practice 10	AI-based dependency scanning		1		
Practice 11	AI-driven incident response and remediation	0			
Practice 12	AI for secure development training			2	
Outcome of appraisal for AI-driven cybersecurity mitigation model for secure software coding level-4 (PA1: malware in codebase) practices covered by the company		Score		1	
		Mitigation level		Understanding	

Level 5	Continuous improvement				
PA1	Poor error handling				
Practice 1	Error classification models			2	
Practice 2	Automated vulnerability scanning			2	
Practice 3	Dynamic runtime monitoring		1		
Practice 4	Predictive code analysis				3
Practice 5	Natural language processing (NLP) bots			2	
Practice 6	AI-driven test case generation			2	
Practice 7	Automated code review				3
Practice 8	Adaptive learning systems			2	
Practice 9	Self-healing code frameworks	0			
Practice 10	Error handling standards enforcement				3
Outcome of appraisal for AI-driven cybersecurity mitigation model for secure software coding level-5 (PA1: poor error handling) practices covered by the company		Score		2	
		Mitigation level		Improvement	

Level 5	Continuous improvement				
PA 2	Supply chain attacks				
Practice 1	AI-powered dependency analysis				3
Practice 2	Automated risk scoring for components		1		
Practice 3	Real-time supply chain monitoring			2	
Practice 4	AI-driven threat intelligence		1		
Practice 5	Behavioral analysis of components				3
Practice 6	Automated patch management		1		
Practice 7	Provenance verification			2	
Practice 8	Threat simulation and resilience testing			2	
Practice 9	Blockchain and AI integration		1		
Practice 10	Policy enforcement with AI	0			
Outcome of appraisal for AI-driven cybersecurity mitigation model for secure software coding level-5 (PA2: supply chain attacks) practices covered by the company		Score		1	
		Mitigation level		Understanding	

Level 5	Continuous improvement				
PA 3	Insider threats				
Practice 1	AI-based user behavior analytics (UBA)		1		
Practice 2	Role-based access control with AI	0			
Practice 3	AI-driven privilege escalation detection			2	
Practice 4	Automated threat detection models		1		
Practice 5	Continuous monitoring and alerts				3
Practice 6	AI-augmented employee risk profiling			2	
Practice 7	Sentiment analysis on communication		1		
Practice 8	AI-powered anomaly detection	0			
Practice 9	Dynamic access and authentication systems		1		
Practice 10	Education and awareness programs		1		
Outcome of appraisal for AI-driven cybersecurity mitigation model for secure software coding level-5 (PA3: Insider Threats) Practices covered by the Company		Score		1	
		Mitigation level		Understanding	

Table 14 gives an illustration of the evaluation spreadsheet.

Through a case study, we evaluated the AI-driven cybersecurity Mitigation Model for Secure Software Coding within a security network organization. The results of the analysis of this organization using the proposed model are presented in Table 16. This table evaluates five levels of an AI-driven cybersecurity Mitigation Model for secure software coding across three key aspects: Levels, Median Scores, and the corresponding Appraisal of the software development organization. Following is the detailed breakdown:

- Levels Overview
  - Level-1: Ad hoc/Uncontrolled
    - Median Score:3
    - Appraisal: Advanced
    - Analysis: Despite being in an early-stage category, the software development organization achieved an Advanced appraisal, indicating that foundational security practices are effectively implemented. Organizations apply AI technologies to locate coding security weaknesses and take appropriate actions at equal priority. The techniques depend on informal approaches because they don't use clear guidelines or efficient distribution methods.
  - Level-2: Planned and Tracked
    - Median Score: 3
    - Appraisal: Advanced
    - Analysis: The secure software development organizations demonstrated planned and tracked software security efforts at this level. At the advanced level, organizations focus on letting AI systems spot threats and abnormal system behavior. Setting up security measures creates a foundation to support ongoing advanced stages of maturity.
  - Level-3: Standardized Processes
    - Median Score: 3
    - Appraisal: Advanced
    - Analysis: The core focus is keeping all business systems uniform across all units. This phase shows that our organization uses AI correctly through established industry techniques:
      - Dependency Scanning
      - Vulnerability management
      - Secure Frameworks: This consistency ensures scalability and fosters long-term security resilience.
  - Level-4: Metrics Driven
    - Median Score: 3
    - Appraisal: Advanced
    - Analysis: Measurable metrics help the organization improve, but an appraisal reveals performance goals that need adjustment:
      - Insufficient use of AI for advanced monitoring and incident response.
      - Lack of established benchmarks to measure and optimize AI-driven security efforts. Focusing on refining metrics and using data insights can elevate this level to advanced maturity.
  - Level-5: Continuous Improvement
    - Median Score: 3
    - Appraisal: Advanced
    - Analysis: As the organization starts using performance indicators, it demonstrates initial continuous improvement understanding and faces challenges deploying AI improvements at scale. The organization finds it challenging to choose practical path actions because performance measurements reveal performance weaknesses:
      - Limited AI adoption in dynamic threat modeling or real-time threat responses.
      - A simple process exists to make security methods better. Advanced AI systems and training will assist the organization in transforming its essential knowledge into thorough security enhancements.
- Key Observations:
  - Advanced Security at Initial Levels: The organization's high appraisals in the first three levels indicate strong foundational and standardized practices.
  - Decline in Later Levels: The major decrease in ratings at the Improvement and Understanding levels shows that our organization needs to strengthen its investment in security metrics while updating its systems continuously.
  - AI as a Differentiator: Early-level success indicators show how well AI tools support secure programming while collecting threat data and constantly monitoring.
- Recommendations for Improvement:
  - Refine Metrics and KPIs (Level 4): Implement measurable security metrics for evaluating AI effectiveness, such as:
    - Time to detect/respond to threats.
    - The number of vulnerabilities patched through AI automation.
  - Expand Continuous Improvement Efforts (Level 5):
    - Incorporate adaptive AI systems for real-time monitoring and predictive threat modeling.
    - Establishing a feedback mechanism to learn from past security incidents and adjust AI-driven processes accordingly.
  - Enhance Training and Awareness:
    - Investment in training developers to align their practices with AI tools, especially at Levels 4 and 5.
- Conclusion

**Table 16:** Case study evaluation of software development organization.

Levels	Five categories of AI-driven cybersecurity mitigation model for secure software coding	Median	Appraisal of software development organization
Level 1	Ad hoc/Uncontrolled	3	Advanced
Level 2	Planned and tracked	3	Advanced
Level 3	Standardized processes	3	Advanced
Level 4	Metrics driven	2	Improvement
Level 5	Continuous improvement	1	Understanding

The software development organization demonstrates strong foundational capabilities in implementing AI-driven cybersecurity practices, as reflected by the advanced appraisals in Levels 1–3. However, its effectiveness declines in Levels 4 and 5, signaling the need for a greater focus on data-driven decision-making and continuous innovation to maintain resilience against evolving cybersecurity threats.

### Hybrid ANN-ISM model effectiveness

The hybrid ANN-ISM model’s effectiveness stems from the synergy between the ANN’s ability to predict cybersecurity risks based on large datasets and ISM’s capacity to reveal the structural relationships between secure software coding. ISM helps us understand cybersecurity risks better so the ANN model can focus on the main risks to predict in software coding.

Combining ISM with the predictive model helps developers understand which threats affect their entire system while making their model better at risk predictions. Interoperability between different security systems lets decision-makers make effective network defense plans. The case study shows that combining predictive accuracy from ANN with interoperability using ISM yields a successful method for improving secure software coding security. The upcoming research will further the model's performance by ensuring it works well with more cybersecurity threats while also making the model completely operational in real-time development settings.

## Implications of the study

This study presents significant theoretical, practical, and policy-related implications. The proposed framework not only facilitates secure development but also provides a powerful, adaptive, and intelligent defense by combining Artificial Neural Networks (ANN) with the Interpretive Structural Modeling (ISM) paradigm to enhance cybersecurity. This study has the following implications:

- Theoretical implications:
  - AI and Cryptology: We hope, with our study, to contribute to this field of Artificial neural networks (ANN) and show that through the use of ANN, it is possible to predict, detect, and mitigate cyber threats in an ever-evolving software development lifecycle
  - Progress in Cybersecurity Methodologies: With the addition of ISM, we get an organized framework that helps illustrate the layers of inner workings of cybersecurity-related problems, thereby facilitating the planning of combating efforts.
  - Broader ANN Application: Leveraging ANN into cybersecurity for software development broadens the scope of the ANN, which was traditionally applied for tasks such as image recognition and natural language processing. The findings of this study lay the groundwork for non-traditional research on adaptive AI-driven security models.
  - Introduction to Structural Modeling of Risks This is where the role of Interactive Strategy Making comes into play as it helps to provide a more dimensional view of risks, which can aid practitioners in drawing bases to various levels of prevention throughout mitigation.
- Practical implications:
  - Enhanced Cyber Threat Detection and Response: A real-time cyber threat detection of the ANN-ISM paradigm enhances software development environment compromises due to conventional security approaches.
  - Proactive rather than Reactive—Armed with the predictive capability of ANN and a hierarchy of risks with ISM, organizations can address security threats proactively instead of becoming reactive.
  - Automation and Efficiency: The offering system automates the security assessment process, requiring less manual intervention and increasing efficiency in secure software development lifecycles (SDLC).
  - Self-Learning Mechanisms: Due to the ever-changing nature of cyber threats, this framework features self-learning systems that adapt to future security obstacles, which lends itself to prolonged adaptability.
  - Scalability and Customization: The organizations can customize the framework to meet their cybersecurity requirements, allowing for a more scalable and adaptable security solution across different s/w dev environments.
- Industry and software development practices implications:
  - Enhanced Secure Development Practices: Integrating ANN-driven cybersecurity across the phases of the software development life cycle (SDLC) fortifies DevSecOps principles, establishing a security-first culture in software development.
  - Cyber Security Cost Reduction: AI automation reduces the reliance on manual processes, thus lowering the cost of cyber security operations in Software Companies.
  - Regulatory Compliance: With a structured approach to addressing security vulnerabilities, ISM helps organizations comply with regulatory requirements such as GDPR, ISO 27001, and NIST Cybersecurity Framework.
  - Optimizing Response to Cyber-Backlash: With predictive analytics at the heart of ANNs, an organization can optimize its response mechanisms to incidents.
  - Fostering Holistic Security Strategy: The study’s findings allow cybersecurity professionals, software developers, and AI engineers to collaborate across disciplines, thus contributing to a more holistic approach to security.
- Policy and governance implications:
  - Cybersecurity Policy: The ANN-ISM framework becomes a reference for policymakers when drawing strong cyber security policies for software development industries for enabling AI-driven security enforcement.
  - Ethical AI in Security: The research emphasizes implementing ethical AI in a security context, calling for transparency and accountability within AI-based threat mitigation decision-making.
  - Standardization of AI in Cybersecurity: With the emergence of AI-driven security models, regulatory authorities may consider the standardization of ANN-based cybersecurity protocols during the software development life cycle.
  - International Cybersecurity Expertise: The findings of this study can also contribute to the formulation of international cybersecurity frameworks, especially in ensuring the security of AI-influenced software across international boundaries.

The impact of this study highlights the transformative potential of AI-enabled cybersecurity within the realm of software development. Thus, the proposed framework not only improves the identification of cyber threats and reduces risks by using ANN and ISM but also provides a systematic and scalable way to administer security. The discoveries will help further academic research and provide profound implications for industry, leading to more AI-centric security wealth of thought. It has the potential to configure multi-cloud and hybrid applications accordingly, plugging gaps in your defense strategy and creating adaptive systems.

## Conclusion and future work

This study improves how well software companies maintain security during software development procedures by integrating ANN with ISM to create an AI-driven solution that detects security weaknesses and mitigates cybersecurity threats. AI has effectively identified vulnerabilities early in the software development lifecycle, preventing threats before they reach the final product. The ANN-ISM framework tracks security risks, detects code injection vulnerabilities, secures against XSS attacks, and prevents real-time buffer overflow incidents. Combining machine-learning systems with security protocols delivers adaptive cybersecurity protection across development platforms. The investigation shows why security should become a fundamental part of every stage of software development rather than an added-on element. Early implementation of cybersecurity strategies enables us to produce better-secured software systems while saving time and money from fixing vulnerabilities after deployment. While the ANN-ISM framework presents a promising solution, further research is needed to improve its applicability and performance. Further projects should explore its integration into DevOps and continuous deployment tools across the development lifecycle.

Future research should focus on:

- Integration with other AI models: Future research could investigate opportunities to integrate ANN with other AI paradigms, including but not limited to reinforcement learning and genetic algorithms, to develop cybersecurity capabilities further.
- Cross-domain applications: The ANN-ISM paradigm could be extended to other domains such as cloud computing security, IoT security, and Blockchain-based cybersecurity frameworks.
- Implementation and validation in practice: Future work needs to adapt and evaluate this framework in practice, integrated into software development workflows.

Our findings significantly impact higher education and industry professionals by introducing a novel AI-driven approach to secure software development. The framework enhances cybersecurity by automating threat detection, reducing unauthorized access risks, and improving vulnerability management through ANN-based learning. Its cross-domain applicability extends to cloud security, IoT, and mobile application development, contributing to secure coding practices while improving cost efficiency and scalability. Moreover, its adoption could influence cybersecurity policies and regulatory frameworks. While the findings illustrate the potential of AI technology in enhancing cybersecurity, limitations such as data availability and quality, model complexity, generalization to diverse software environments, performance overhead, adaptability to evolving threats, and validation/testing challenges must be acknowledged. Future research should focus on enhancing the framework's adaptability to various security contexts and expanding its applicability across different scenarios further to strengthen software defense in our increasingly digital world.

## Limitations of the study

This research is the first of its kind to provide a new framework for AI-based cybersecurity protection in software development using ANN and ISM paradigms, and certain limitations need to be mentioned for better transparency and balanced discussion of the applicability, effectiveness as well as scalability of the presented model.

- Data availability and quality: The performance of the proposed framework is mainly dependent on the quality, diversity, and size of the dataset used to train the ANN model. The testing dataset used in the current study was similar to that employed in many publicly available cybersecurity repositories and specific enterprise case studies. Nevertheless, dataset selection, its non-uniqueness, and CDU (Completeness, Diversity, and Uniqueness) issues could potentially affect the model's generalizability to real-world applications in various industries.
- Generalization and scalability of the model: While the ANN-ISM framework was validated in emulated software development environments, its applicability in diverse programming paradigms, software architectures, and cybersecurity threats remains to be seen. The model has its strengths and weaknesses, and its effectiveness in detecting and mitigating threats can depend on factors like The programming language deployed (Java, Python, C++).
- Time and Resources Intensive: High computational workloads and resource-intensive ANN training processes in the context of large software systems merge into the phase of the ANN with ISM. The deep learning part of the framework consumes a lot of processing, memory, and storage power, which can be a barrier to deployment in low-resource environments like start-ups or low-scale software development firms.
- The interpretability and explainability of AI decisions: Commonly, the ANN is powerful for recognizing patterns and trends for cybersecurity threats but mainly operates as a black box, thus having poor interpretability and explainability of the reasoning behind those decisions. Besides introducing ISM for increasing structural clarity, the overall framework might still be complex if specific alerts or vulnerabilities are highlighted for non-coded audiences (compliance managers, solutions managers).
- Reliance on past styles of attack: It is important to note that the ANN-ISM framework relies heavily on past attack patterns and predefined knowledge of the cybersecurity field to identify vulnerabilities and risks. Nonetheless, new emerging attack vectors like AI-generated cyber threats, polymorphic malware, and advanced persistent threats (APTs) might not always be appropriately discovered. The lack of a proper learning mechanism and live updates can render the framework unable to adapt to new and unknown attack techniques.
- Errors with potential false positives and negatives: While the ANN-ISM framework can be an effective intrusion detection system, it may also misclassify certain software behaviors as threats (false positives) or fail to detect subtle but critical vulnerabilities (false negatives) despite being trained on thousands of benign programs. The downside of false positives is unnecessary security alerts, which makes the workload of cybersecurity teams heavier. The most considerable risk is a false negative, which keeps vulnerabilities hidden in the software development pipeline for too long.
- Data Collection Optimization and hyperparameter tuning: Several methods need to be optimized in a way that requires a balance between detection accuracy and the usability of the system.
- Opportunity cost due to limited testing in real-world deployment: While the framework has been validated on synthetic data in experimental conditions, it is still early for widespread deployment in practice. Software development environments and cybersecurity threats are dynamic. Therefore, further investigations are warranted to explore and validate the solution through an empirical study, longitudinal testing, and real-world implementation.
- Ethical and legal implications: Some of the more troubling ethical and legal issues of AI-driven cybersecurity solutions include Data Privacy Issues, Which may expose sensitive codebases and security logs. Regulatory compliance: The framework should ensure compliance with industry-specific cybersecurity regulations (e.g., GDPR, NIST, ISO/IEC 27001).
- AI predictions demonstration of bias: Training of biased data, SAAS application-related threat prediction aspects might not be detected at each software due to inequality leading to fairness and the security type emphasis.
- Not Enough human-in-the-loop validation: The suggested framework is entirely AI-based, requiring little human effort. However, these skilled cyber-threat hunters utilize contextual knowledge to identify advanced cyber threats. Without a HITL, it can lessen the requirements for the framework’s flexibility in managing ambiguous or novel assault patterns that may necessitate expert analysis.

Still, the ANN-ISM framework provides an excellent strategic direction to improve cybersecurity in software development. Future work should further work on models-generalizability, computational efficiency, explainability, and real-time adaptive learning must be watchful to render its application in different software development systems.

## Supplementary Information

Supplementary Information.

## Data Availability

All data generated or analyzed during this study are included in this manuscript.
