# Supplementary material for: AI-driven cybersecurity framework for software development based on the ANN-ISM paradigm
Source: Sci Rep. 2025 Apr 18;15:13423. doi: 10.1038/s41598-025-97204-y (PMC12008409; doi:10.1038/s41598-025-97204-y)
Supplement: Supplementary file 1 — Supplementary Information. [file 41598_2025_97204_MOESM1_ESM.docx]

**Appendix:**

**This research survey includes the following demographic questions about "Identification of Cybersecurity Threats and its AI Practices for Secure Software Coding":**

**1. Age:**

**- What is your age?**

**- [ ] Under 18**

**- [ ] 18–24**

**- [ ] 25–34**

**- [ ] 35–44**

**- [ ] 45–54**

**- [ ] 55–64**

**- [ ] 65 or older**

**2. Gender:**

**- What is your gender?**

**- [ ] Male**

**- [ ] Female**

**- [ ] Non-binary**

**- [ ] Prefer not to say**

**I am choosing the option Other with a specific entry of "Elderly Healthcare Professional" for this field.**

**3. Highest Level of Education Completed:**

**Which level of education have you obtained?**

**- [ ] High School or equivalent**

**- [ ] Some College or Associate Degree**

**- [ ] Bachelor’s Degree**

**- [ ] Master’s Degree**

**- [ ] Doctorate**

**The other option contains an empty field which should be completed as: __________**

**4. Occupation:**

**Presently you hold what position?**

**- [ ] Software Developer/Engineer**

**- [ ] Cybersecurity Specialist**

**- [ ] IT Professional**

**- [ ] Researcher/Academic**

**- [ ] Student**

**I belong to an unknown category which I will specify below: __________**

**5. Years of Experience in Software Development/Coding:**

**Please indicate your years spent developing software code or programming.**

**- [ ] Less than 1 year**

**- [ ] 1–3 years**

**- [ ] 4–6 years**

**- [ ] 7–10 years**

**- [ ] More than 10 years**

**6. Familiarity with Cybersecurity Threats:**

**You should rate the level of your familiarity with standard software development cyber threats.**

**- [ ] Very Unfamiliar**

**- [ ] Unfamiliar**

**- [ ] Neutral**

**- [ ] Familiar**

**- [ ] Very Familiar**

**7. I have worked with AI tools and practices during my experience of developing software for security purposes.**

**Performing any work in software development for cybersecurity purposes with AI tools or practices has been part of your experience.**

**- [ ] Yes**

**- [ ] No**

**8. Size of Organization:**

**What number of personnel work at your current workplace?**

**- [ ] Small (1–10 employees)**

**- [ ] Medium (11–50 employees)**

**- [ ] Large (51–200 employees)**

**- [ ] Very Large (200+ employees)**

**9. Region:**

**Which geographical region do you occupy at present?**

**- [ ] North America**

**- [ ] Europe**

**- [ ] Asia**

**- [ ] Africa**

**- [ ] Latin America**

**- [ ] Australia/Oceania**

**10. Industry:**

**Your organization operates within which industry?**

**- [ ] Information Technology**

**- [ ] Finance/Banking**

**- [ ] Healthcare**

**- [ ] Government**

**- [ ] Education**

**Please specify other options in this empty field: __________.**
